# Genome-Wide Identification and Expression Pattern Profiling of the Aquaporin Gene Family in Papaya (*Carica papaya* L.)

**DOI:** 10.3390/ijms242417276

**Published:** 2023-12-08

**Authors:** Qiuxia Zeng, Haifeng Jia, Yaying Ma, Liangwei Xu, Ray Ming, Jingjing Yue

**Affiliations:** 1 Center for Genomics and Biotechnology, Fujian Agriculture and Forestry University, Fuzhou 350002, China; qxzeng0914@163.com (Q.Z.); jiahaifeng1218@163.com (H.J.); yaying123@yeah.net (Y.M.); xulw332020@163.com (L.X.); 2 College of Agriculture, Fujian Agriculture and Forestry University, Fuzhou 350002, China; 3 College of Life Science, Fujian Agriculture and Forestry University, Fuzhou 350002, China

**Keywords:** papaya, aquaporins, expression pattern, fruit development, abiotic and biotic stress

## Abstract

Aquaporins (AQPs) are mainly responsible for the transportation of water and other small molecules such as CO_2_ and H_2_O_2,_ and they perform diverse functions in plant growth, in development, and under stress conditions. They are also active participants in cell signal transduction in plants. However, little is known about AQP diversity, biological functions, and protein characteristics in papaya. To better understand the structure and function of *CpAQPs* in papaya, a total of 29 *CpAQPs* were identified and classified into five subfamilies. Analysis of gene structure and conserved motifs revealed that *CpAQPs* exhibited a degree of conservation, with some differentiation among subfamilies. The predicted interaction network showed that the PIP subfamily had the strongest protein interactions within the subfamily, while the SIP subfamily showed extensive interaction with members of the PIP, TIP, NIP, and XIP subfamilies. Furthermore, the analysis of *CpAQPs*’ promoters revealed a large number of *cis*-elements participating in light, hormone, and stress responses. *CpAQPs* exhibited different expression patterns in various tissues and under different stress conditions. Collectively, these results provided a foundation for further functional investigations of *CpAQPs* in ripening, as well as leaf, flower, fruit, and seed development. They also shed light on the potential roles of *CpAQP* genes in response to environmental factors, offering valuable insights into their biological functions in papaya.

## 1. Introduction

Aquaporins (AQPs) belong to membrane intrinsic proteins (MIPs) and play a critical role in facilitating the rapid and selective transportation of water and many other small molecules across cell membranes [1]. AQPs are widely found in most species, ranging from bacteria to higher eukaryotes [2]. Based on differences in sequence homology and subcellular localization in different subfamilies, AQPs can be classified into five subfamilies, namely, plasma membrane intrinsic proteins (PIPs), tonoplast intrinsic proteins (TIPs), nodulin26-like intrinsic proteins (NIPs), small basic intrinsic proteins (SIPs) and X-intrinsic proteins (XIPs), in higher plants [3]. However, there are seven subfamilies in *Physcomitrella patens*, including five of the subfamilies mentioned above and a further two AQP subfamilies: hybrid intrinsic proteins (HIPs) and GlpF-like intrinsic proteins (GIPs). This indicates that the diversification of AQPs is an early event and that HIP and GIP subfamilies have been lost in higher plants [4]. In addition to HIPs and GIPs, the XIP subfamily was lost in monocots and the dicot family Brassicaceae [5].

Generally, there are more subtypes of AQPs in plants than in animals and bacteria, and the number of AQPs is usually higher in plants than in animals [6]. For instance, 120 AQPs consisting of 43 PIPs, 35 TIPs, and 31NIPs, and 11 SIPs were identified in canola [7], and 72 AQPs, including 22 PIPs, 23NIPs, 17 NIPs, 8 SIPs, and 2 XIPs were found in soybean [5]. The smallest number, 19 AQPs, was found in the moss *Selaginella moellendorffii* with 3 PIPs, 3 NIPs, 8 NIPs, 1 SIPs, and 3 XIPs [5]. Additionally, AQPs have been identified in many species, such as *A. thaliana* [8], cucumber [9], melon [10], *Medicago* [11], grape [12] cherry [13], rice [14], and *Kandelia obovata* [15].

AQPs have a highly conserved structure, consisting of six transmembrane (TM) helices; these are connected by five loops, named Loop A (LA) to E (LE), with two conserved asparagine–proline–alanine (NPA) motifs distributed in LB and LE. Additionally, there is an aromatic/arginine (Ar/R) selective filter including residues of Helix 2 (H2), Helix 5 (H5), LE1, and LE2, and Froger’s positions containing residues of P1 to P5 [16,17]. The selectivity with which solutes are transported through AQPs is very complex and is related to the AQPs’ structure. Usually, four AQP molecules form a homotetramer, with each monomer having an independent permeable function. The fifth narrow channel, created by the polymerization of the four monomers and located in the center, may also have substrate permeability [18]. Being hydrophobic, the fifth channel is regarded as a gas transport channel in plants, suggesting that AQPs are not only water channels but also multifunctional channels [19]. Moreover, AQPs are also responsible for the transportation of small molecules that include glycerol, NH_3_, and urea; micronutrients, such as silicon (Si) and boron (B); and signal molecules, such as hydrogen peroxide (H_2_O_2_). In addition to water, they also include reactive oxygen species (ROS) and gases, such as carbon dioxide (CO_2_) and *oxygen* (O_2_) [20,21]. AQPs are tolerant of metals, such as aluminum (Al) [22] and zinc (Zn) [23,24]. Different subfamilies of AQPs show high specificity for different substrates because of their different structures [25]. Furthermore, AQPs are more than simply transporters, as they are not only able to change the transportation of signaling molecules but can also act as active participants in cell signaling pathways. This suggests that, rather than being limited to functioning as water channels, they possess diverse functions and show potential for crop molecular breeding [19].

The structure of AQPs is highly conserved between species within a subfamily but different in different subfamilies, suggesting that the function of AQPs is similar in a subfamily but quite diverse across different subfamilies. In cucumber, *CsAQPs* have been found to play an important role in response to salt stress [9], while *CmAQPs* play a role in water and solute transport in melon [10]. In grape, *VvAQPs* are implicated in various physiological aspects, especially in the process of berry development [12], whereas *PaAQPs* are implicated in the ability to adapt to abiotic stress in cherry [13]. *KoAQPs* in *Kandelia obovata* play diverse roles in plant adaptability [15]. These studies have improved our understanding of AQP structure and function.

In plants, AQPs play versatile roles in growth and development, various physiological processes, and stress tolerance [1]. For instance, the small peptide PEP7 activated AQPs and further affected lateral root development in *A. thaliana*, highlighting the potential role of AQPs in lateral root development [26]. A recent study discovered that the GsCPK16-dependent phosphorylation and activation of *GsPIP2;2* regulate flower opening and re-opening in *Gentiana scabra*, suggesting that phosphorylation is an essential regulatory mechanism for AQPs [27]. AQPs also play a vital role in abiotic stress pathways, such as salt and drought resistance. *TdPIP2;1* overexpression in durum wheat improved germination rates and biomass production under high salt and osmotic stress conditions [28], while *TsPIP1;1* overexpression enhanced salt tolerance in transgenic rice plants [29]. In *Medicago truncatula*, *MtCAS31* promotes the autophagic degradation of *MtPIP2;7*, enhancing drought resistance in plants [30]. In contrast, *RhPIP2;1* interacts with *RhPTM* and regulates the balance of ‘growth and survival’ in rose under drought stress conditions [31]. Additionally, AQPs show great potential in biotic stress responses, including insect and disease resistance. A *Phytophthora sojae* CRN effector, CRN78, mediates the phosphorylation of AQPs, promoting their degradation and ultimately inhibiting aquaporin-mediated immunity. This suggests that AQPs may serve as positive regulators of plant immunity, particularly in response to disease stress [32]. Recent studies have revealed that phosphorylation at two sites of *TaPIP2;10* improves wheat yield and increases resistance to powdery mildew, scab, and aphids. *TaPIP2;10* has also been found to confer resistance to two fungal diseases in wheat [33].

Papaya (*Carica papaya* L.) is a renowned tropical fruit crop celebrated for its nutritional benefits and economic value [34]. It also serves as an exemplary plant system for studying sex chromosome evolution, featuring three sex types: XX for females, XY for males, and XY^h^ for hermaphrodites [35,36]. In regions of papaya production, the predominant threat is the papaya ring spot virus (PRSV), transmitted by aphids. Developing PRSV-resistant transgenic papaya would be the most effective strategy for PRSV prevention [37].

Considering the potential of AQPs to influence plant growth, development, and resistance to biotic and abiotic factors, it is imperative to explore and comprehend the functions of AQPs in papaya. However, knowledge regarding the diversity of papaya’s AQPs, their biological functions, and the proteins’ characteristics remains elusive. Fortunately, recent advances in the genome sequencing of two *C. papaya* genomes have paved the way for a comprehensive analysis of papaya gene families [38,39].

To better understand the structure of AQPs and their potential biological functions in papaya, 29 *CpAQP* genes were successfully identified in the SunUp papaya genome. Comprehensive analyses were conducted, encompassing their chromosomal locations, collinearity, classification, evolutionary relationships, networks of interactions with different subfamilies, gene structures, conserved motifs, domains, and expression patterns in different tissues and developmental stages. This research serves as a foundation for future investigations into the physiology and functionality of the AQP family in papaya. It will enrich our understanding of the AQP family in papaya, facilitating the discovery and screening of candidate genes aimed at enhancing biotic and abiotic tolerance. It, therefore, opens up avenues for harnessing the application potential of AQP genes in papaya.

## 2. Results

### 2.1. Identification, Phylogenetic Analysis, and Characterization of CpAQPs

**Figure 1:**
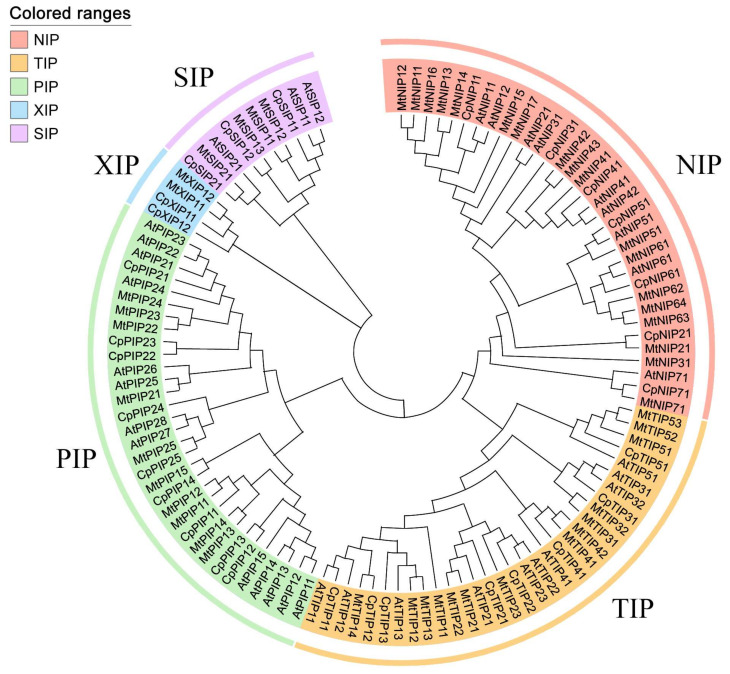
Phylogenetic tree of AQPs from *A. thaliana*, *M. truncatula*, and *C. papaya*. PIP, TIP, NIP, SIP, and XIP represent plasma membrane intrinsic proteins, tonoplast intrinsic proteins, NOD26-like intrinsic proteins, small basic intrinsic proteins, and x-intrinsic proteins, respectively. Different subfamilies were represented by different colors, namely, green, yellow, red, purple, and blue.

A HMMER search identified 29 CpAQP candidate genes in papaya. For consistency with other plant species in terms of nomenclature, names were assigned to the papaya AQPs, and a phylogenetic analysis was conducted (Table 1, Figure 1). We performed multi-sequence alignment analyses and constructed a phylogenetic tree with *C. papaya*, *A. thaliana*, and *M. truncatula*. We classified the CpAQPs into five subfamilies: nine PIPs, eight TIPs, seven NIPs, three SIPs, and two XIPs (Figure 1, Table S1). Within the PIP subfamily, we identified two subgroups, namely, PIP1 and PIP2, while the TIP family exhibited five subgroups, TIP1 to TIP5. Additionally, the NIP subfamily consisted of seven subgroups, each containing one member. The SIP subfamily was divided into two subgroups, SIP1 and SIP2, while XIP comprised a single group (Table S1).

Similar to the AQPs in *A. thaliana*, the PIP subfamily was also the most abundant in papaya. The XIP subfamily, however, was found in papaya but was absent in *A. thaliana*. Although papaya and *Medicago* shared the same AQP subfamilies and subgroups, there were variations in the number of members within these subfamilies. Specifically, the NIP subfamily was the largest in *Medicago*, while the PIP subfamily was the largest in papaya.

In-depth analyses of the properties of CpAQPs revealed a range of characteristics. The protein sequences varied in length, spanning 225 to 322 amino acids. Their molecular weights (MWs) ranged from 23,867.33 to 34,726.58, while the isoelectric points (pIs) exhibited variations from 4.83 to 10.42 (Table 1). Within the TIP subfamily, all members pIs were lower than 7, except for CpTIP5;1. Conversely, in the other subfamilies, all members had pIs higher than 7, except for CpNIP3;1 (Table 1).

The instability indices of most of the CpAQPs were less than 40, indicating relative stability; the exception was CpNIP4;1 (47.11), which was considered unstable (Table 1). The aliphatic index varied from 92.83 to 119.27. The grand average of the hydropathy (GRAVY) values varied from 0.327 to 1.063, indicating that CpAQPs were hydrophobic. Among the subfamilies, the PIP subfamilies exhibited the lowest average GRAVY value (0.44), while the TIP subfamilies had the highest average GRAVY value (0.77), indicating that TIPs were more hydrophobic, likely contributing to their increased water permeability compared to PIPs.

Predictions of sub-cellular localization indicated that all members of the TIP subfamily were located in the vacuole, with the exception of CpTIP5;1, which was located in both the cell membrane and vacuole. Meanwhile, other AQP members were predominantly localized in the cell membrane, except for CpNIP4;1, CpNIP7;1, and CpSIP1;1, which were found in both the cell membrane and vacuole (Table 1). These results underscore the diversity of the sub-cellular localization of CpAQPs, reflecting the wide array of functions they may possess. Furthermore, the TM domain prediction revealed that 23 of 29 CpAQPs included 6 TM domains, whereas the remaining 6 CpAQPs had between 4 and 7 predicted TM domains (Table 1).

### 2.2. Chromosomal Locations and Collinearity Analysis of CpAQPs

**Figure 2:**
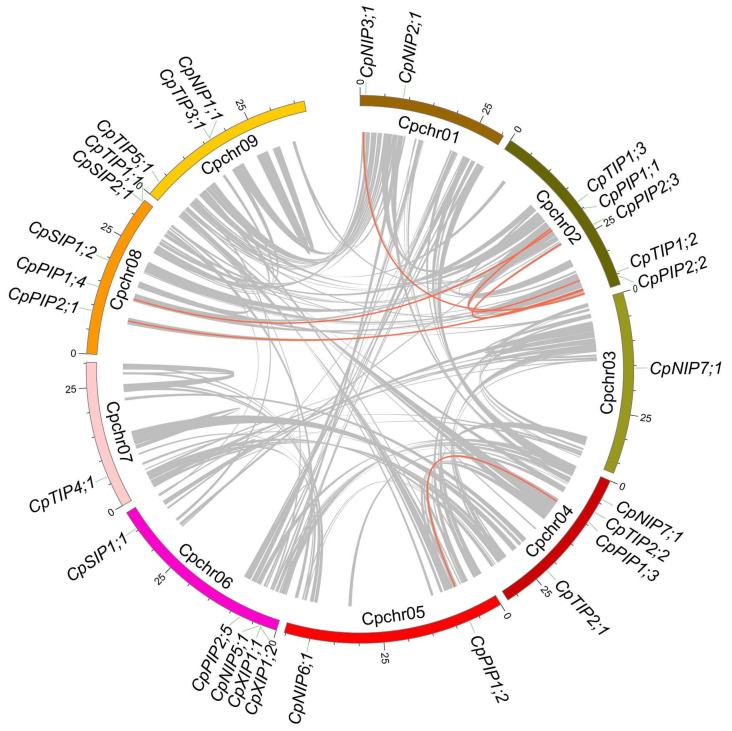
Chromosomal locations of CpAQP genes and their synteny analysis in papaya. Gene pairs with syntenic relationships were linked by gray lines, and the gene pairs with syntenic relationships of CpAQPs were linked with red lines.

Twenty-nine CpAQP genes were distributed across nine chromosomes. CpAQPs were most abundant on Chr 2, with six genes, and least abundant on Chr 3 and 7, both with only one gene (Figure 2). Syntenic analysis of the *C. papaya* genome was performed to identify relationships among the CpAQP genes, and six collinearity gene pairs were identified (Figure 2). The Ka/Ks ratios of these six collinearity gene pairs were all less than 1 (Table 2). Among them, the lowest Ka/Ks ratio was only 0.0280 (CpPIP1;3–CpPIP1;2), while the highest was 0.1310 (CpNIP3;1–CpNIP4;1). The results indicate that these six collinear gene pairs are under purifying selection.

### 2.3. Analyses of Gene Structures, Motifs, and Domains of CpAQPs

**Figure 3:**
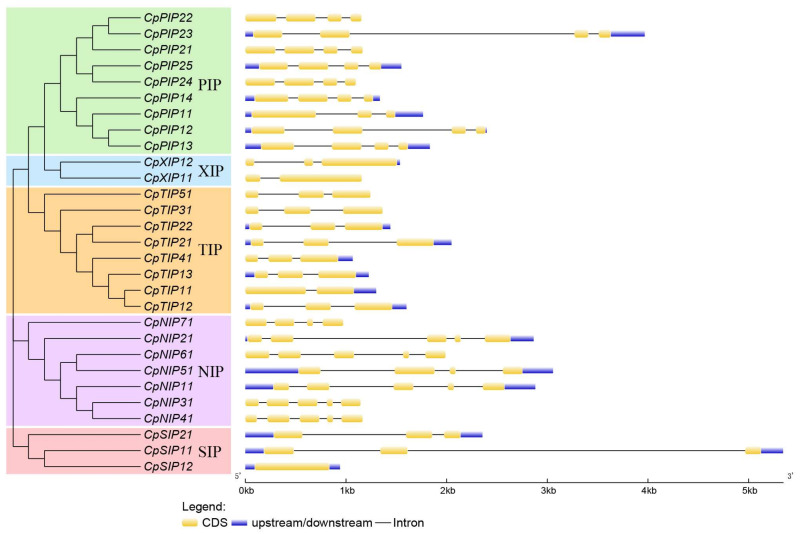
Analysis of CpAQP gene structure in papaya. The phylogenetic tree of CpAQPs was constructed using the neighbor-joining method in MEGA (v X) with 1000 replicates. Gene structures of all CpAQP genes were visualized using the GSDS program. The yellow boxes represented exons, the lines represented introns, and the blue boxes represented up-steam or down-steam of untranslated region (UTR) regions.

Analysis of the CpAQP gene structures revealed intriguing patterns. The number of exons ranged from one to five (Figure 3). Notably, most PIP subfamily members had four exons, with the exception of CpPIP1;1, which possessed three exons. Similarly, most TIP subfamily members contained three exons, except for CpTIP1;1, which had two exons. Among the NIP subfamily, five of seven members had five exons, while CpNIP5;1 and CpNIP7;1 had four exons.

**Figure 4:**
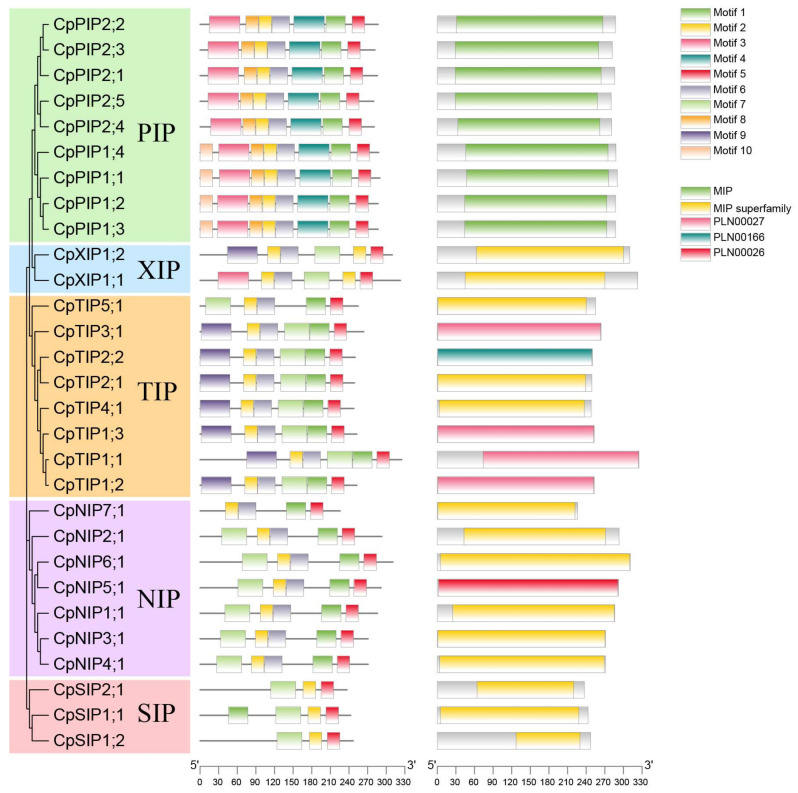
Analysis of the conserved motifs and domains of CpAQP proteins in papaya. The MEME tool was utilized for the motif analysis using the protein sequence. Ten conserved motifs were set and visually distinguished by different colored boxes. Additionally, the conserved domains of CpAQPs were identified using NCBI CDD, and different conserved domains were distinguished using varied colored boxes. The motifs and conserved domains were visualized using TBtools (v1.098769).

Motifs analysis revealed the presence of 10 conserved motifs (Motifs 1 to 10) in all CpAQPs, with most CpAQP proteins sharing similar motifs in the same subfamily (Figure 4). For example, the PIP subfamily comprised seven conserved motifs, with Motif 10 being unique to the PIP2 subgroup. Within the TIP subfamily, six conserved motifs were identified, except for CpTIP5;1, which lacked Motif 9. In the NIP subfamily, five conserved motifs were present, with CpNIP7;1 being the exception. Interestingly, Motif 2 was found in all CpAQPs, highlighting its widespread conservation. Analysis of conserved domains revealed that all members of the PIP subfamily harbored the MIP conserved domain, which is indicative of their common functionality (Figure 4).

### 2.4. The Conserved and Substrate-Specific Residues in CpAQPs

AQPs are primarily recognized for their role in transporting small molecules. Specific amino acid domains and residues within AQPs determine their selectivity in transporting substrates. Key determinants include the two NPA motifs, the Ar/R filter, and Froger’s positions, which are crucial for the physiological functions of AQPs [40]. A multiple sequence alignment analysis was conducted to identify these crucial CpAQP features (Table 3, Figure S1). These features play a vital role in substrate selectivity within CpAQPs. Notably, both the PIP and TIP subfamilies, as well as the majority of the NIP subfamily members, displayed two conserved NPA motifs in LB and LE. The third residue of the first NPA motif was serine (S) in CpNIP5;1. The second NPA motif had valine (V) in CpNIP5;1 (Table 3). However, the third residue of the first NPA was not conserved in the SIP and XIP subfamilies; this residue exhibited variations, such as threonine, serine, leucine, valine, and isoleucine.

The amino acid residues of CpAQPs comprised phenylalanine (F), histidine (H), threonine (T), arginine (R), glycine (G), isoleucine (I), cystine (C), serine (S), leucine (L), tyrosine (Y), tryptophan (W), methionine (M), glutarnine (Q), lysine (K), glutamic acid (E), and valine (V). The Ar/R selectivity filter sequence F–H–T–R was highly conserved in the PIP subfamily, whereas in the TIP subfamily, subgroups, such as TIP1, exhibited F–I–A–V, and TIP2 had F–I–G–R, except for A–I–A–R in CpTIP4;1 and S–F–G–C in CpTIP5;1. In contrast, the Ar/R selectivity filter sequence displayed the highest diversity in the NIP subfamily, with variations across its seven subgroups.

Regarding Froger’s positions, the P1 position varied among different subfamilies, while the P2 position remained well conserved in the PIP, TIP, and NIP subfamilies but not in the SIP and XIP subfamilies. The P3, P4, and P5 positions were well-conserved in various CpAQP subfamilies. For example, P3, P4, and P5 residues were A–F–W in the PIP and XIP subfamilies and A–Y–W in the TIP and SIP subfamilies. However, P5 positions were not conserved in the NIP subfamily, with different members exhibiting residues, such as I, L, V, and M.

Phosphorylation and dephosphorylation play crucial roles in regulating the permeability of membranes and, consequently, the function of AQPs [41]. Identifying the phosphorylation sites within AQPs represents the initial step in understanding the regulatory mechanisms governing their phosphorylation. An analysis was performed to predict the number and types of phosphorylation sites within 29 CpAQP proteins. Among them, 28 CpAQPs exhibited all three types of phosphorylation sites, including serine (S), threonine (T), and tyrosine (Y), while CpSIP2;1 had only two types of phosphorylation sites, S and T, with no Y phosphorylation site (Table 3). The number of phosphorylation sites among the 29 CpAQP proteins ranged from 14 to 59. In most CpAQP proteins, the number of S phosphorylation sites exceeded those of T and Y phosphorylation sites. Furthermore, the PIP, NIP, and XIP subfamilies had substantially more phosphorylation sites than the SIP and TIP subfamilies (Figure S2 and Table S2).

### 2.5. Protein–Protein Interaction Network of CpAQPs

**Figure 5:**
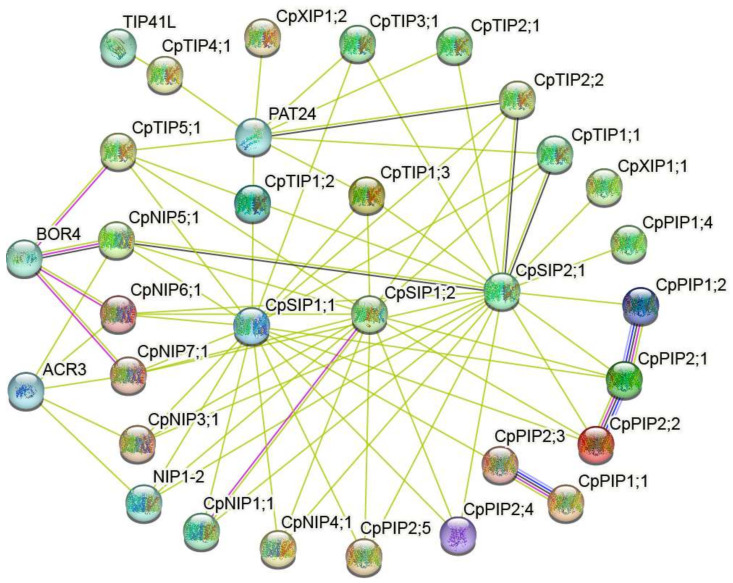
Protein interaction network of CpAQPs in papaya based on the STRING database. The nodes represented CpAQP proteins, and proteins interacted with CpAQP proteins. The edges represented protein–protein associations, including known interactions, predicted interactions, and other interactions. Among them, the different lines, including the pink line, red line, black line, yellow-green line, dark blue, and light blue, indicated experimentally determined gene fusions, co-expression, text mining, gene co-occurrence, and protein homology, respectively.

Previous studies have shown that AQPs interacts not only within and between members of AQP subfamilies but also with other proteins, as discovered in wheat, passion fruit, and apricot [42,43,44]. To gain further insight into the interaction of AQP member proteins in papaya, CpAQPs were selected for the construction of the interaction network for prediction purposes and based on the orthologs of the model plant *A. thaliana* (Figure 5). It became clear that 27 CpAQPs had an orthologous relationship with *A. thaliana*, and 5 other proteins were predicted to interact with different subfamilies of the CpAQP proteins.

Compared with other subfamilies, the PIP subfamily members exhibited the strongest protein interactions within the subfamily. CpPIP1;2, CpPIP2;1, and CpPIP2;2 also had a strong interaction, as did CpPIP1;1 and CpPIP2;3, indicating that members of the PIP1 and PIP2 subfamilies interacted with each other and then functioned in an indirect manner. Several TIP subfamily members had robust interactions with Protein S-acyltransferase 24 (PAT24), a member of the DHHC palmitoyltransferase family. Additionally, some members of the NIP subfamily interacted with both Boron transporter 4 (BOR4), part of the anion exchanger (TC 2.A.31.3) family, and ACT domain-containing protein (ACR3), considered as new regulator or sensor proteins.

CpTIP4;1 had interactions with the TIP41-like protein, suggesting that this may play a role in the regulation of the target of rapamycin (TOR) signaling pathway. Furthermore, members of the SIP subfamily exhibited extensive interactions with other AQP subfamily members, suggesting that further investigation into their potential role is required. These results demonstrate that the protein–protein interaction network provides the basis for further CpAQP function research; the results also provide candidate genes and new insights for future investigation.

### 2.6. Molecular Modeling of CpAQPs

**Figure 6:**
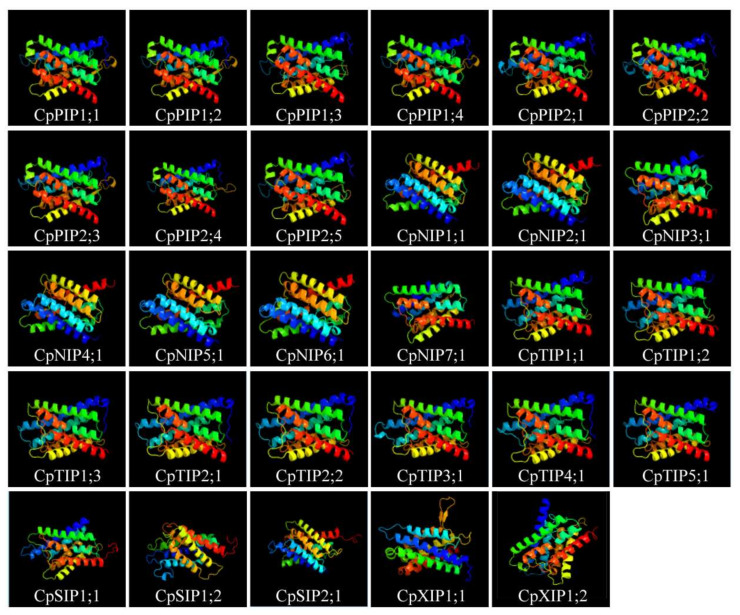
Predicted three-dimensional (3D) models of CpAQP proteins. The Phyre2 online program was used to predict the 3D models of all CpAQP proteins. All the models were colored a rainbow from the N to C terminus.

Molecular dynamics simulation is a valuable tool for comprehending the permeation mechanism of AQPs and plays an important role in understanding their substrate specificity [45]. Three-dimensional (3D) protein models of all CpAQPs were predicted with a confidence level of 100%. The predicted residue coverage ranged from 69% to 98% (Table S2). Notably, the majority of CpAQPs consisted of six TM helices (Figure 6). Additionally, predictions for the secondary structure of all CpAQPs were conducted; the α-helix contributed between 55% and 73% of CpAQPs, while β-strands were only detected in CpNIP2;1 (1%) and CpTIP1;1 (2%). TM helices ranged from 45% to 59% (Table S3). Ramachandran plot analysis revealed that the conformation of all protein models was consistent with the rules of stereochemistry, with amino acid residues falling in the most favored regions and additional allowed regions accounting for more than 90% of the total protein. Furthermore, 10 protein models were regarded as good quality models with over 90% of residues in the most favored regions (Table S4). These results indicated that the 3D protein model predictions were quite precise and would provide an important basis for the protein functional analysis of CpAQPs in papaya.

### 2.7. Analysis of Cis-Acting Elements in CpAQPs

**Figure 7:**
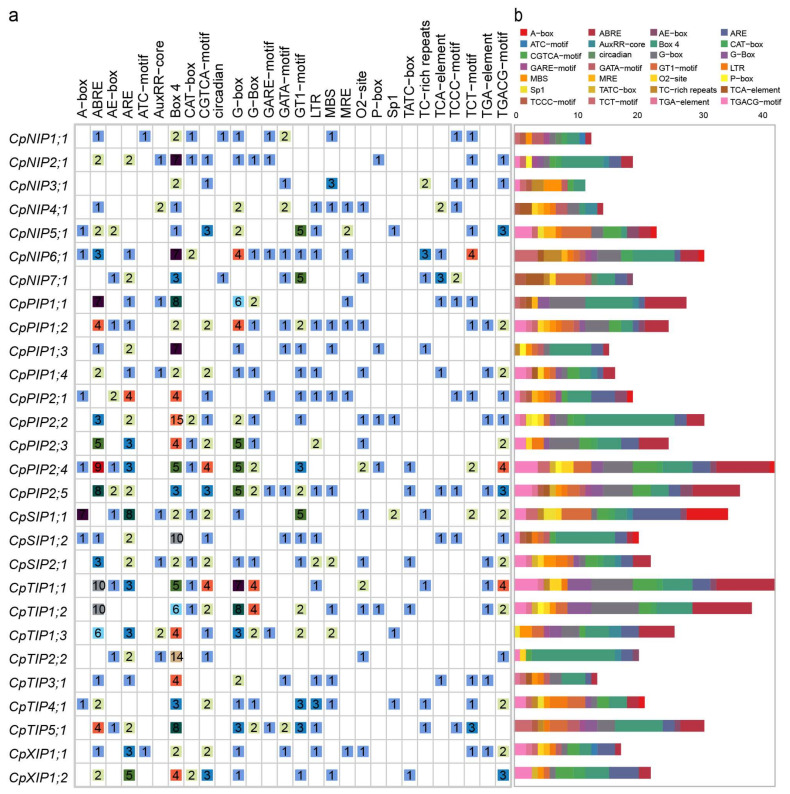
Analysis of *cis*-acting element numbers in papaya *CpAQP* genes. (**a**) The different colors and numbers of the grid indicated the numbers of different promoter elements in these *CpAQP* genes. (**b**) The different colored histogram represents the sum of the *cis*-acting elements in each category.

Recently, a study characterizing the *cis*-acting elements within the grapevine AQP family predicted potential transcription factors, including AP2/ERF, bZIP, NAC, and R2R3-MYB. This discovery indicates that specific transcription factors regulate particular AQP genes by binding to the *cis*-acting elements [46]. To identify putative *cis*-acting elements in the promoter region of *CpAQPs*, 2000 bp upstream of the promoter regions of these *CpAQPs* were analyzed. Twenty-eight elements were identified in the promoter regions, including light, hormone, and stress-related *cis*-regulatory elements (Figure 7). The light-related *cis*-regulatory elements identified in the *CpAQP* promoter included Box-4, G-Box, TCT-motif, and MRE. Notably, the Box-4 element was the most abundant, being present in all CpAQP promoter regions. For example, the promoters of *CpPIP2;2* and *CpTIP2;2* contained 15 and 14 Box-4 elements, respectively.

Hormone-related *cis*-regulatory elements were also found in the promoter regions; these included 88 abscisic acid (ABA)-responsive elements (ABRE), 10 auxin-responsive elements (AuxRR-core), 7 gibberellin-responsive elements (GARE-motif), 40 MeJA-responsive elements (CGTCA-motif), and 11 salicylic acid-responsive elements (TCA-element). These elements were present in 23, 8, 7, 20, and 8 *CpAQP* promoters, respectively, indicating that *CpAQPs* could respond to hormonal regulation, especially ABA.

Stress-related *cis*-regulatory elements, including 11 defense and stress-responsive elements (TC-rich repeats), 55 anaerobic induction elements (ARE), 17 drought-inducibility elements (MBS), and 19 low-temperature responsive elements (LTR), were found in 8, 22, 13, and 15 *CpAQP* promoters, respectively. Notably, AREs were present in all the members of the PIP, SIP, and XIP subfamilies but absent in some members of the NIP and TIP subfamilies. These results indicate that most *CpAQPs* play essential roles in coping with abiotic and biotic stress. Moreover, the circadian element was detected only in the promoter regions of *CpNIP1;1* and *CpNIP7;1*. This indicates that *CpAQPs* may function in response to the biotic and abiotic stress and may also be involved in the processes of growth and development in papaya.

### 2.8. Expression Profile of CpAQP Genes in Different Tissues

**Figure 8:**
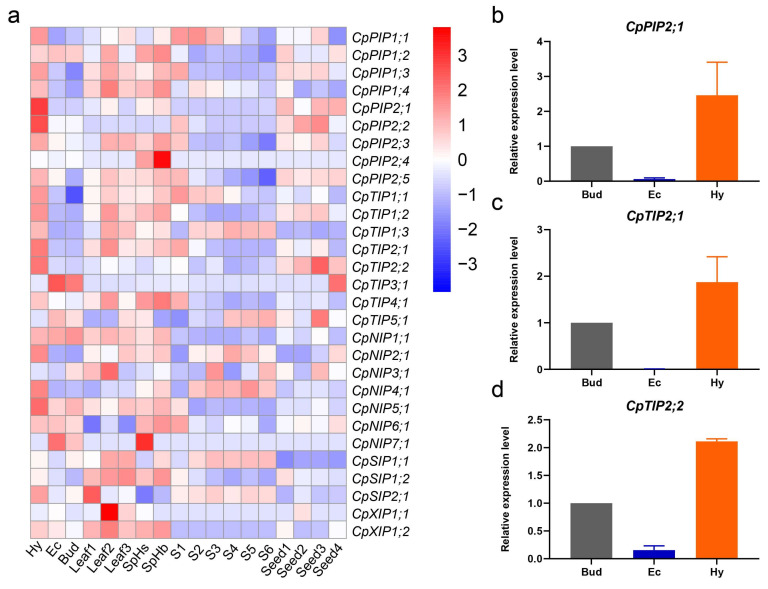
Expression pattern profiling of *CpAQP* genes in papaya by RNA-seq analysis and RT-qPCR analysis. (**a**) Expression profiles of *CpAQP* genes in different stages and different tissues. Hy, Ec, and Bud represented the hypocotyl, embryonic callus, and regeneration buds. Leaf1, Leaf2, and Leaf3 indicated very young leaf, young leaf, and mature leaf, and SpHs and SpHb indicated the hermaphrodite flower that was less than 1.5mm and 8mm, respectively. S1–S6 indicated immature green, color break, 25% yellow, 50% yellow, 75% yellow, and 100% yellow, respectively. Seed1–4 represented 7, 15, 31, and 65 days after pollination. The blue color represented low expression, and the red color represented high expression. The average FPKM of three biological replicates was used as the expression level, and they were normalized by Log2 (FPKM + 1). (**b**–**d**) Real-time quantitative PCR (RT-qPCR) analysis of the expression patterns of *CpPIP2;1*, *CpTIP2;1*, *CpTIP2;2* in the hypocotyl, embryonic callus and regeneration bud.

AQP genes have been reported to play versatile roles in the processes of plant growth and development. They facilitate vegetative and reproductive growth by transporting various small molecules [47]. AQP genes exhibit distinct expression patterns across different tissues, indicating the diversity of their functions. To explore the diverse roles of *CpAQPs* in C. papaya, the expression patterns of *CpAQP* genes in different tissues were analyzed, including hypocotyl (Hy), embryogenic callus (Ec), regenerative buds (Bud), two flower stages (SpHs and SpHb), three leaf stages (stages 1–3), six fruit stages (S1–S6), and four seed stages (Stages 1–4) (Figure 8a). Clearly, *CpAQP* genes were expressed in at least one tissue, with most members displaying high expression in the hypocotyl, flower, leaf, first stage of fruit development, and third stage of seed development (Figure 8a). During callus induction and bud regeneration processes, *CpPIP2;1*, *CpPIP2;2*, and *CpTIP2;2* showed higher expression in the hypocotyl, while *CpTIP3;1* and *CpNIP7;1* exhibited high expression in embryogenic calli and regenerative buds. Interestingly, nearly all *CpAQPs* were expressed in flowers, with a high expression in SpHb.

Most *CpAQP* genes were expressed throughout all three leaf-development stages, with higher expression in Stage 2. In the case of the SIP1 subgroup genes, these displayed high expression in Stages 2 and 3 but low expression in Stage 1, while the SIP2 subgroup genes exhibited higher expression in Stage 1 and lower expression in Stages 2 and 3. During fruit ripening, most *CpAQP* genes had abundant expression in the S1 stage compared with the other five stages. However, members of the XIP subfamily showed no expression at any stage. Notably, two genes (*CpPIP2;5* and *CpTIP1;1*) belonging to the PIP and TIP subfamilies displayed extremely high expression in the S1 stage and were subsequently down-regulated in S2–S6. In contrast, *CpSIP1;1* showed increased expression during fruit development.

Additionally, during seed development, AQP genes with high expression were primarily found in the PIP and TIP subfamilies, while the other subfamilies showed lower or no expression. These expression patterns showed that most *CpAQPs* were expressed in different stages of different tissues, suggesting that *CpAQPs* are active in various stages of different tissues and indicating their presence and function in nearly all plant tissues.

To validate the RNA-seq data, three genes (*CpPIP2;1*, *CpTIP2;1* and *CpTIP2;2*) and three tissues (Hy, Ec, and Bud) were selected for qPCR analysis (Figure 8b–d). As shown in the figure, the expression patterns for the different tissues in these three genes were consistent with the results of RNA-seq analysis.

### 2.9. Expression Profiles of CpAQP Genes under Biotic and Abiotic Stress

During their growth and development, plants are exposed to a variety of biotic stressors, including bacteria, viruses, fungi, and insects, and abiotic stressors, such as drought, extreme temperatures, salinity, and nutritional imbalance [48,49]. To predict the potential roles of AQPs in coping with abiotic and biotic stress in papaya, the responses of *CpAQPs* to low temperature and PRSV stress were observed. The expression levels of all 29 *CpAQP* genes were assessed under low temperature and PRSV treatment.

**Figure 9:**
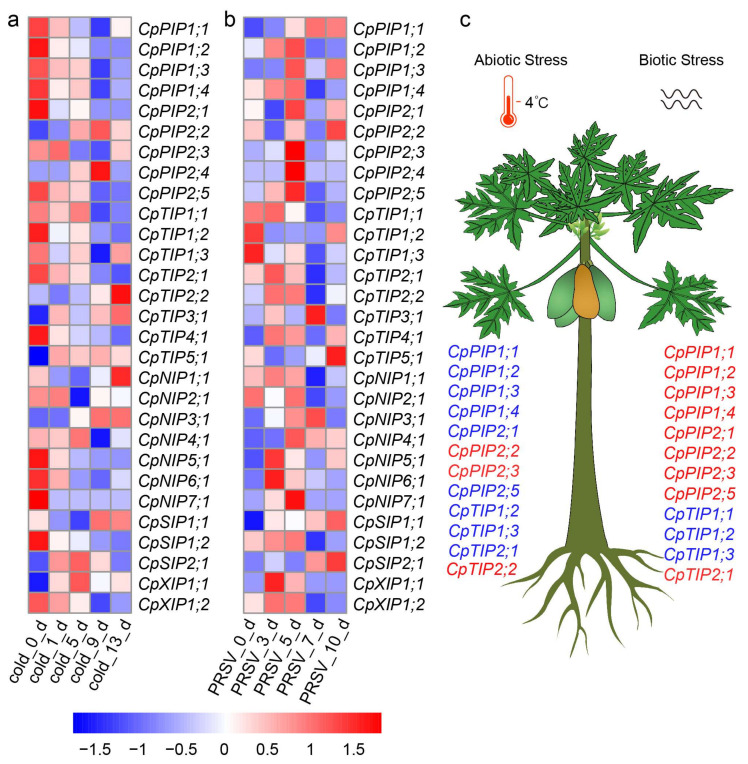
Expression pattern profiling of *CpAQP* genes in papaya under cold and virus stress. (**a**) Expression profiles of *CpAQP* genes responding to cold stress. (**b**) Expression profiles of *CpAQP* genes responding to PRSV infection. (**c**) Schematic models for expression patterns of *CpAQPs* in response to abiotic and biotic stress. cold_0_d represented no cold treatment, and papaya seedlings planted under 28 °C were taken as a control, while cold_1_d, cold_5_d, cold_9_d, cold_13_d represented papaya seedlings were treated at 4 °C for 1, 5, 9, and 13 days, respectively. PRSV_0_d, PRSV_3_d, PRSV_5_d, PRSV_7_d, and PRSV_10_d represented that papaya seedlings were infected by PRSV for 0, 3, 5, 7, and 10 days, respectively. The blue color represents low expression, and the red color represents high expression. The average FPKM of three biological replicates were used as expression levels, and they were normalized by Log2 (FPKM + 1). *CpAQPs* marked red indicated the up-regulated genes, while the blue color indicated the down-regulated genes.

As the duration of low-temperature treatment increased, the expression of most AQP genes was down-regulated. However, *CpPIP2;2* and *CpPIP2;4* showed an initial increase followed by a decrease, with the highest expression level observed on the ninth day after low-temperature treatment. In contrast, *CpTIP2;2* and *CpNIP1;1* reached their highest expression levels on the 13th day of cold treatment (Figure 9a). When compared with the early stage (0 days) of papaya plants subjected to PRSV infection, the expression levels of most *CpAQP* genes increased and then decreased with the duration of PRSV treatment. However, two members of the CpTIP1 subfamily (*CpTIP1;2* and *CpTIP1;3*) were down-regulated (Figure 9b). These findings collectively suggested that most *CpAQPs* were actively involved in responding to cold treatment and PRSV infection in papaya, albeit in different ways (Figure 9c).

## 3. Discussion

Increasingly available plant genomes have enabled the identification and characterization of the AQPs in many plant species, laying a foundation for functional studies. For instance, 35 AQPs were found in *A. thaliana* [8], 33 in rice [14], 46 in *Medicago* [11], 39 in cucumber [9], 38 in pomegranate [50], 42 in apple [51], 33 in pineapple [52], 47 in banana [53], 27 in passion fruit [43], and 33 in pitaya [54]. The diverse functions of AQPs have been demonstrated, for example, in regulating plant development and growth and responding to abiotic stress, such as drought, cold, and salt resistance. Additionally, AQP genes play a potential role in responding to biotic stress, such as disease and insects [33,55].

In a previous study, 28 *CpAQPs* were identified, and *CpNIP2;1* was regarded as a Si transporter [5]. However, the detailed information about AQPs in papaya is still lacking. Here, 29 CpAQPs were identified and divided into five subfamilies, including nine PIPs, eight TIPs, seven NIPs, three SIPs, and two XIPs (Figure 1). A previous study concluded that GIPs and HIPs were lost in the evolutionary process [3]. The XIP subfamily was also absent in species of monocots and some species of dicots, such as *A. thaliana* and *Brassica rapa*, which belong to Brassicaceae. Based on a comparative analysis of AQPs in 25 plant species, it has been suggested that species harboring the XIP subfamily may be more ancient [5]. To date, there have been few functional studies on XIP subfamily members, and their functions require further elucidation.

Analysis of pI showed that members of the XIPs subfamily were acidic, except for *CpTIP5;1*, which is located in the cell membrane and vacuole, while other members of this subfamily were neutral or alkaline, except for *CpNIP3;1* (Table 1). This phenomenon was also found in several other species, such as banana [53], cucumber [9], and melon [10]; this may be related to the sub-cellular localization of AQPs. Members of the TIP subfamily were mainly located in the vacuole, while members of other AQP subfamilies were located in the cell membrane (Table 1). Previous studies have found that most plant cytoplasm is neutral or slightly alkaline, while most vacuoles are acidic, suggesting that the difference in protein acidity and alkalinity may be closely related to subcellular localization and biological functions [56].

AQPs in plants can regulate the process of water uptake and transport in many ways; post-translational modifications, including phosphorylation, glycosylation, deamidation, acetylation, methylation, and ubiquitination, are the most important gating regulations [1,41]. The phosphorylation of protein modifications is the most common type. A previous study showed that *GsCPK16* catalyzed phosphorylation of S280 and participated in the activation of *GsPIP2;2*; it also regulated the opening of flowers in *Gentiana scabra* [27]. In *Rhsa hybrida*, a water deficit resulted in phosphorylation of the S273 serine residue and ultimately affected plant growth [31]. In *Triticum aestivum*, *TaPIP2;10* increased the photosynthesis and defense capacity of wheat by phosphorylation at S280 and S121, respectively [33]. A recent study indicated that the *SbAT1* gene enhanced the salt-alkali tolerance of sorghum by inhibiting the phosphorylation of SbPIP2s [57]. Collectively, these studies indicated that the phosphorylation of AQPs is essential in plant growth and development and in response to biotic and abiotic stress. Analysis of phosphorylation in CpAQPs revealed that 28 of 29 CpAQPs were present in all three types of phosphorylation sites, the S phosphorylation site being the most abundant in most CpAQPs, except for CpPIP1;2 (Table 3). This indicates that phosphorylation of the S site may play an important role in the regulation of AQP activity in papaya.

Similarly, as observed in sweet orange [58] and cucumber [9], the gene structure analysis of papaya also revealed that the numbers of exons and introns were highly specific for each subfamily (Figure 3), suggesting functional conservation in each subfamily. The motifs were also conserved in all CpAQPs, except for some motifs that were specific to each subfamily (Figure 4). NPA motifs, Ar/R selectivity, and Froger’s positions are considered important for the transportation of substrate and the functions of AQPs. For instance, putative Si transporters have been confirmed based on the presence of two NPA domains with 108 aa spacing, GSGR selectivity filters, and different Froger’s residues from 985 AQPs [5]. However, our study showed that the Ar/R selectivity filters were T–S–G–R, which is different from the findings of the previous study (Table 3).

Protein S-acylation has been shown to be an important means of regulating lipid post-translational modification of proteins, playing a vital role in plant growth and development and in response to various adversities [59]. In *A. thaliana*, *PAT24* was reported to be involved in root hair growth, and *PAT10* was reported to respond to plant development and salt stress [60,61]. Our results demonstrate that many CpTIPs interacted with PAT24 (Figure 5), suggesting that some CpTIPs may function by interacting with PAT24. Another study revealed that *ACR3* can improve tolerance to arsenic stress in *A. thaliana* [62]. Additionally, *OsNIP1;1* and *OsNIP3;3* can decrease arsenic accumulation by overexpression in rice [63]. Our interaction network predicted that CpNIP3;1, CpNIP5;1, CpNIP6;1, and CpNIP7;1 may interact with ACR3 in papaya (Figure 5), indicating that a novel regulatory mechanism for arsenic tolerance may exist in papaya through the interaction of CpNIPs and CpACR3. A study showed that the transcript levels of several PIPs would be decreased under boron toxicity to prevent boron excess accumulation, while another study revealed that *NIP5;1* is essential for efficient boron uptake [64,65]. The predicted interaction network for papaya demonstrates that CpTIP5;1 and the other three members of CpNIPs interacted with BOR4 (Figure 5), indicating that CpAQPs may play an indirect role in the process of boron uptake and accumulation. In sugar beet, NIPs have been reported to play important roles in silicon uptake and growth [66]. These studies reveal that NIPs play a role in the absorption, transport, and accumulation of arsenic, boron, and silicon. TIP41L may be involved in the regulation of the target of rapamycin (TOR) signaling pathway and could interact with CpTIP4;1; this indicates the potential roles of CpAQPs during the growth, development, and metabolism of papaya.

Promoters containing abundant *cis*-acting elements were considered important regulators of gene expression [67,68]. In soybean, the activity of the *GmTIP2;6* promoters was strongly induced under heat stress and l-aminocyclopropane-l-carboxylic acid (ACC) treatment in transgenic plants [69]. In banana, the promoter functions of *MaTIP1;2* [70] and *MaPIP1;1* [71] were implicated in response to drought and salt stress and in response to drought stress inducibility, respectively. Analysis of the promoter regions in *CpAQP* genes revealed that there were abundant *cis*-elements involved in light, hormone, and stress responses, indicating the potential functions of *CpAQPs* involved in papaya growth and development and stress responses.

It is widely accepted that water uptake and TM water flow in roots are mainly regulated by members of the PIP and TIP subfamilies located in the plasma membrane and the tonoplast of the plant cells; these are the most abundant compared with other AQP subfamilies in most plant species [72]. AQPs not only play an important role in water transportation but also transport small molecules in plants. Water transport is essential for many processes, including plant growth and development, seed germination and development, flower opening and re-opening, rapid fruit expansion, and ripening. Gene expression patterns are closely related to gene functions. Several genes, namely *CpPIP1;1*, *CpPIP1;2*, *CpPIP1;3*, *CpPIP2;3*, *CpPIP2;5*, *CpTIP1;1*, *CpTIP2;1*, and *CpTIP4;1*, showed high expression levels in at least one tissue (Figure 8a), suggesting that members of the PIP and TIP subfamilies play an important role in different tissues. Among them, four genes, including *CpPIP1;3*, *CpPIP2;3*, *CpPIP2;5*, and *CpTIP1;1*, showed high expression in the S1 stage of fruit development and ripening, while *CpPIP1;1* showed high expression in the S1 and S2 stages (Figure 8a). This indicates that these genes might be involved in regulating the development and ripening of papaya fruit, especially in the rapid expansion of fruit during early development. In strawberry, *FaPIP1;1* was highly expressed in fruit, but there was low or no expression in other tissues. The expression pattern of *FaPIP1;1* was also associated with auxins and with the ripening process [73]. A study on strawberry revealed that *FaNIP1;1*, located in the plasma membrane and regulated by abscisic acid and auxins, could be involved in the water accumulation of strawberry ripening [74]. Effects of PIP and TIP members on fruit development and ripening have also been found in tomato [75], grape [46], and passion fruit [43], showing the same expression patterns as in papaya.

Our study revealed that members of the PIP and TIP subfamilies showed high expression in seed development. A recent study demonstrated that when *GmPIP2;9* was highly expressed in roots and developing seeds, it enhanced drought resistance and promoted seed development in soybean [76]. PIP1 has been reported to promote water absorption in the process of seed water uptake in pea [77]. These results suggest that the functions of the PIP and TIP subfamilies may be conserved during fruit development and ripening and seed development in different species. Additionally, the expression levels of NIP members, such as *SlNIP2;1* and *SlNIP6;1*, increased from the first stage to the last stage of fruit development in tomato, suggesting that these two genes may play a vital role in fruit development [75]. In pineapple, *AcNIP5;1* was highly expressed in the white basal part of the leaf and was considered critical for sufficient boron uptake and water regulation [52]. In pitaya, *HuNIP6;1* showed a high expression in the Os 3 stage of the petals and sepals and was further confirmed to be involved in the flowering process [54]. The expression profiles of *CpAQPs* in flowers and at different stages of leaf and fruit development revealed that most members of the NIP subfamily showed a lower expression in these tissues except for *CpNIP7;1*, which was not expressed in any tissues (Figure 8a). These results suggest that members of the NIP subfamily were functionally differentiated in different species during the evolution process.

There was no expression of any members of the XIP subfamily in any stage of fruit. *CpXIP1;1* was not expressed in flowers or leaves; in contrast, *CpXIP1;2* was highly expressed in flowers and leaves, especially in leaf stage 2, which differed from the XIPs in rubber trees. Of the three members of the XIP subfamily, only *HbXIP2;1* was expressed in all vegetative tissues [78]. *CpXIP1;2* was expressed in vegetative tissues and reproductive organs (flowers), indicating that XIPs may have different functions in papaya.

For the three members of the SIP subfamily, there were several patterns *CpSIP1;2* showed high expression in leaves and flowers and low expression in all other tissues. *CpSIP1;1* showed increasing expression during leaf and fruit development and in fruit ripening, and *CpSIP2;1* was highly expressed in the hypocotyl and the earliest stage of leaf development. These patterns illustrate the diversity of SIPs’ functions.

Researchers have proposed 15 unknowns in plant abiotic stress, one of them being, ‘How can one aquaporin have so many roles in a plant?’ [79], indicating the potential function of AQPs in abiotic stress. In *Puccinellia nuttalliana,* the transcription levels of several PIP genes were up-regulated under salt stress, indicating that these genes play important roles in responding to salt stress [80]. Another study revealed that PLATZ4 inhibited the gene expression of *PIP2;8*, improving drought resistance in *A. thaliana* [81]. These studies indicate the important role of AQPs in abiotic stress. Additionally, protein kinase SnRK2.4, which is a key regulator, works by phosphorylating the first cytoplasmic loop of the PIP protein and further regulates the water transport capacity of roots in *A. thaliana* [82]. MePIP2;7 is involved in magnesium ion transport through its interaction with MeMGT9 in cassava [83], suggesting that AQP genes have great potential for transporting small molecules. Another investigation revealed that AQPs not only function as transporters but are also involved in the process of cell signal transduction [19]. A recent study found that the Sugarcane mosaic virus (SCMV) regulates H_2_O_2_ transport by targeting ScPIP2;4 to establish infection, indicating that the AQP gene serves a function in the process of virus infection [84]. Our results regarding the expression of CpAQP changes in papaya after virus infection and the specific mechanism of action require further clarification (Figure 9a,b).

Our findings have improved our understanding of AQPs’ structure and function and laid a foundation for the further study of *CpAQP* function in papaya; they also provide important candidate genes for the molecular breeding of resistant papaya.

## 4. Materials and Methods

### 4.1. Identification of Candidate AQP Genes in the Papaya Genome

The updated papaya genome version was used for analysis [39]. To identify candidate AQP genes, an HMM search was performed against the new *C. papaya* genome using the MIP domain (PF00230), which was downloaded from InterPro (https://www.ebi.ac.uk/interpro/entry/pfam/PF00230/curation/, accessed on 20 October 2022). BLASTP (v2.11.0+) [85] search was also performed with 35 AtAQPs protein sequences in *A. thaliana* as queries [8], and the cutoff of E-value was set to e^−5^. The two results were merged and submitted to NCBI CDD (https://www.ncbi.nlm.nih.gov/Structure/bwrpsb/bwrpsb.cgi, accessed on 20 October 2022) to further confirm the MIP domain.

### 4.2. Chromosomal Locations of CpAQPs and Collinearity Analysis in Papaya

The chromosomal distributions of *CpAQP* genes were displayed using the Circos (v 0.69-8) software. MCScanX [86] and BLASTP (v2.11.0+) [85] software were used to identify collinear blocks in the *C. papaya* genome and scan the collinearity gene pairs of *CpAQP* genes. The synonymous (Ks) and nonsynonymous (Ka) substitution rates of the gene pairs were calculated using ParaAT2.0 [87], and the Ka/Ks ratios < 1 indicate negative or purifying selection, while Ka/Ks ratios = 1 represent neutral selection and Ka/Ks ratios > 1 means positive selection.

### 4.3. Protein Sequence Alignment and Phylogenetic Analysis

Based on multiple sequence alignment and a phylogenetic tree constructed with clearly classified AQPs from *A. thaliana* [6] and *M. truncatula* [9], the candidate CpAQPs were classified into different subfamilies and subgroups and then were named. Multiple sequence alignments were performed using ClustalW [88], and the phylogenetic tree was constructed using MEGA (v X) [89] software with the neighbor-joining method and 1000 bootstrap replicates.

### 4.4. Protein Properties, Prediction of Subcellular Localization and Transmembrane Helical Domains in Papaya

The protein properties of CpAQPs, including amino acid length, isoelectric point (pI), molecular weight (MW), instability index, aliphatic index, and the grand average of hydropathicity (GRAVY) were predicted using the ExPASY program (https://web.expasy.org/protparam/, accessed on 25 October 2022) [90]. The subcellular localizations of CpAQPs were predicted using the Plant-mPLoc (v 2.0) software [91]. TMHMM-2.0 (https://services.healthtech.dtu.dk/service.php?TMHMM-2.0, accessed on 25 October 2022) was used to predict the transmembrane helices in CpAQP proteins. Conserved residues of the NPA domain, Ar/R selectivity filter (H2, H5, LE1, and LE2), and Froger’s positions (P1–P5) were identified using multiple sequence alignment with ClustalW [88] of the different subfamilies of CpAQPs and the multiple sequence alignment result was displayed using ESPript 3.0 [92]. The prediction of protein phosphate sites was performed using the NetPhos 3.1 Server (http://www.cbs.dtu.dk/services/NetPhos/, accessed on 27 October 2022).

### 4.5. Gene Structure, Motif Composition, and Domains Analysis in Papaya

The coding sequences (CDSs) of *CpAQPs* and their corresponding genomic sequences were used to predict gene structures, and the results were displayed using the Gene Structure Display Server (GSDS2.0, http://gsds.cbi.pku.edu.cn/, accessed on 4 October 2022) [93]. The protein sequences of CpAQPs were submitted to the MEME Suite 5.4.0 program (https://meme-suite.org/meme/tools/meme, accessed on 4 October 2022) to identify the conserved motifs. The conserved domain of CpAQPs was identified using NCBI CDD (https://www.ncbi.nlm.nih.gov/Structure/bwrpsb/bwrpsb.cgi, accessed on 20 October 2022). A schematic diagram of the conserved motifs and domains was visualized with the TBtools (v 1.098769) software [94].

### 4.6. Interaction Network Construction of CpAQPs

The STRING (http://string-db.org/cgi, accessed on 8 August 2023) database was used to predict the interaction network of CpAQPs based on their orthologs in the model plant *A. thaliana*. The minimum required interaction score was set to 0.70, and the maximum number of interactors was set to no more than 5.

### 4.7. Prediction of Secondary and 3D Structures

The secondary structure of 29 CpAQPs protein sequences was predicted using the Phyre2 server (http://www.sbg.bio.ic.ac.uk/phyre2/html/page.cgi?id=index, accessed on 14 October 2022) with E-value < 0.001. Then, 29 CpAQPs protein sequences were also submitted to the Phyre2 server (http://www.sbg.bio.ic.ac.uk/phyre2/html/page.cgi?id=index, accessed on 14 October 2022) to predict the three-dimensional (3D) structures under ‘Normal’ mode based on the homologous modeling. Subsequently, the predicted protein models were confirmed using the PROCHECK [95] program in the SAVER v6.0 (https://saves.mbi.ucla.edu/, accessed on 24 November 2023) through the Ramachandran plot analysis with the protein database file (Protein Data Bank, PDB format) generated by Phyre 2.

### 4.8. Promoter Cis-Acting Element Analysis

The 2000-bp upstream sequences of the translation initiator codon of the *CpAQP* genes were obtained by TBtools (v 1.098769) [94], and then they were submitted to the PlantCARE (http://bioinformatics.psb.ugent.be/webtools/plantcare/html/, accessed on 11 October 2022) database for the prediction of candidate *cis*-acting regulatory elements in the *CpAQP* promoter sequences. The distribution of these candidate *cis*-acting regulatory elements was visualized using the R (v 4.0.1) package.

### 4.9. Expression Analysis of RNA Data

To investigate the expression pattern of *CpAQPs* in different papaya tissues and different stages in papaya fruit, the fragments per kilobase of transcript per million mapped reads (FPKM) values for each *CpAQP* gene were extracted from the expression data from previously published RNA-seq data [39], including three stages of leaf (very young leaves from 4-month-old papaya represented stage 1, while young leaves from 9-month-old mature hermaphroditic papaya plants collected from the top represented stage 2, and mature leaves from 9-month-old mature hermaphroditic papaya plants collected from the bottom were considered as stage 3), two stages of flower (small flower and big flower: the size of flower was less than 1.5 mm and 8 mm, respectively), six stages of fruit. The six stages of fruit were immature green (S1), color break (S2), 25% yellow (S3), 50% yellow (S4), 75% yellow (S5), and 100% yellow (S6). Four stages of seed were obtained from a 9-month-old mature hermaphroditic papaya plant. The expression data that included hypocotyl (Hy), embryogenic callus (Ec), and regenerative buds were published previously [96]. The Sequence Read Archive (SRA) accession number of these published RNA-seq data is presented in Table S5. RNA-seq data from four stages (7, 15, 31, and 65 days after pollination) of seed development were also collected to explore the CpAQPs’ roles in the development of papaya seeds (unpublished data). Other RNA-seq data including the cold treatment of papaya leaves from 4-month-old Zhongbai plants (4 °C treatment for 0 d, 1d, 5 d, 9 d, 13 d) and virus infection of papaya leaves from 4-month-old Hongling plants (PRSV treatment for 0 d, 3 d, 5 d, 7 d, 10 d) were collected to study the expression of *CpAQPs* in response to abiotic and biotic stress (unpublished data) and these data were provided by Chuang Ge and Xi Chen in our lab, respectively. Detailed information on these data is presented in Table S6. The sequencing platform was Illumina HiSeq TM 2500 (Illumina, San Diego, CA, USA). All samples had three biological replicates except for leaf, flower, and fruit. The heat maps of the *CpAQP* genes were drawn using the R package based on their expression levels.

The SunUp genome assembly was downloaded from the NCBI Genome database under the accession number JAIUCH000000000. The raw RNA-seq data published previously were available from the NCBI Sequence Read Archive (SRA) database under Bioproject PRJNA727683 and PRJNA1043857. All the raw RNA-seq data were developed using quality control with fastp (v0.23.4) [97] to get the clean reads, and then these clean reads were mapped into the reference genome of SunUp using HISAT2 (v2.2.1) [98]. StringTie (v2.1.6) was used for quantitation [99].

### 4.10. Primer Design, RNA Extraction and Quantitative Real-Time PCR

Three *CpAQP* genes were selected for RT-PCR detection, including one member of the PIP subfamily and two members of TIP subfamily, respectively. The primers of the selected *CpAQP* genes were designed based on the coding region (CDS) using Primer3 Plus online software (https://www.primer3plus.com/index.html, accessed on 20 October 2022) [100]. The primer sequences for *CpActin2*, as provided by Zerpa-Catanho et al. [101], were used as internal controls (Table S7). All primers were listed with *CpActin2*.

Total RNA was extracted from hypocotyls and leaves using the MiniBEST Universal RNA Extraction Kit (TaKaRa, Japan, Tokyo), and reverse transcription reactions were performed using PrimeScript™ 1st Strand cDNA Synthesis Kit (TaKaRa, Japan, Tokyo) according to the manufacturer’s protocols. qPCR was performed with SYBR^®^ qPCR Master Mix in a 20 μL reaction system, and an ABI Prism 7500 sequence detector (Applied Biosystems, Foster City, CA, USA) was used for qPCR programmer running. Finally, the quantitative results of qRT-PCR were calculated using the 2^−ΔΔCT^ method [102].

## 5. Conclusions

AQP proteins play critical roles in various developmental processes and plant responses to various biotic and abiotic stresses. In this study, 29 *CpAQPs* were identified and divided into five subfamilies, including PIPs, TIPs, NIPs, SIPs, and XIPs in the *C. papaya* genome. Chromosomal locations of *CpAQPs* and collinearity analysis showed that *CpAQPs* were unevenly distributed on the chromosomes and that six pairs of collinear genes underwent purification selection. Protein properties, subcellular localization, transmembrane helical domains, gene structures, 3D structures, *cis*-acting elements in promoter regions, and interaction network of *CpAQPs* were analyzed in detail, revealing the differences and diversification of AQPs subfamily structure and function. Additionally, the expression files of *CpAQPs* in different tissues and development stages and under biotic and abiotic stress made us better understand the potential roles of *CpAQPs* in papaya growth and development and the process of coping with biotic and abiotic stress. Interestingly, *CpAQPs* exhibit responsiveness to both biotic and abiotic stresses, such as PRSV and cold, suggesting that *CpAQPs* may play important roles in enhancing plant stress resistance. These results will lay a foundation for a better understanding of the essential functions of *CpAQP* genes and will contribute to the breeding of papaya, particularly those focused on enhancing papaya stress resistance. Furthermore, the mechanism underlying *CpAQPs*’ response to stress resistance requires further elucidation in future studies.

## Figures and Tables

**Table 1:** Nomenclature and protein properties of CpAQPs in papaya.

Gene ID	Name	Length (aa)	MW	pI	Instability Index	Aliphatic Index	GRAVY	TM	Location
*sunup02G0011550*	CpPIP1;1	289	30,785.79	8.6	30.3	96.92	0.367	6	Cell membrane
*sunup05G0008140*	CpPIP1;2	286	30,825.86	8.86	28.19	92.83	0.327	6	Cell membrane
*sunup04G0010360*	CpPIP1;3	296	30,639.70	8.84	29.04	97.62	0.392	6	Cell membrane
*sunup08G0003790*	CpPIP1;4	287	30,796.95	9	32.1	99.65	0.426	6	Cell membrane
*sunup08G0001380*	CpPIP2;1	285	30,452.26	8.54	34.68	97.58	0.396	6	Cell membrane
*sunup02G0026030*	CpPIP2;2	286	30,498.46	7.7	31.1	103.74	0.512	6	Cell membrane
*sunup02G0015860*	CpPIP2;3	281	29,994.92	9.03	32.45	98.65	0.554	6	Cell membrane
*sunup04G0005660*	CpPIP2;4	280	29,782.66	8.33	37.2	99.68	0.474	6	Cell membrane
*sunup06G0008910*	CpPIP2;5	279	29,696.7	9.25	33.37	103.23	0.508	6	Cell membrane
*sunup08G0020690*	CpTIP1;1	324	33,787.65	5.87	31.91	95.86	0.468	7	Vacuole
*sunup02G0024580*	CpTIP1;2	252	25,871.99	5.38	29.75	109.68	0.874	7	Vacuole
*sunup02G0009620*	CpTIP1;3	252	26,199.31	5.78	25.12	101.51	0.754	6	Vacuole
*sunup04G0016050*	CpTIP2;1	248	25,167.61	6	33.04	119.27	1.063	7	Vacuole
*sunup04G0008810*	CpTIP2;2	249	25,088.20	4.83	27.65	117.5	0.975	6	Vacuole
*sunup09G0013480*	CpTIP3;1	263	27,897.60	6.97	38.64	113.8	0.593	5	Vacuole
*sunup07G0007260*	CpTIP4;1	247	26,050.50	6.27	23.77	115.63	0.774	6	Vacuole
*sunup09G0004720*	CpTIP5;1	254	26,179.32	7.86	37.41	99.09	0.654	6	Cell membrane, Vacuole
*sunup09G0013540*	CpNIP1;1	285	30,548.43	8.77	33.65	100.95	0.489	6	Cell membrane
*sunup01G0009560*	CpNIP2;1	292	31,050.14	8.94	34.28	104.59	0.424	6	Cell membrane
*sunup01G0001680*	CpNIP3;1	270	28,363.81	5.35	27.44	102.22	0.589	6	Cell membrane
*sunup02G0027710*	CpNIP4;1	270	29,376.49	8.43	47.11	113.63	0.656	6	Cell membrane, Vacuole
*sunup06G0005050*	CpNIP5;1	291	30,154.16	8.78	34.26	101.99	0.535	5	Cell membrane
*sunup05G0019830*	CpNIP6;1	310	32,296.47	8.71	37	97.65	0.424	6	Cell membrane
*sunup03G0016950*	CpNIP7;1	225	23,867.33	8.27	30.02	114.84	0.997	6	Cell membrane, Vacuole
*sunup06G0021920*	CpSIP1;1	242	25,821.78	9.52	27	112.15	0.856	6	Cell membrane, Vacuole
*sunup08G0007580*	CpSIP1;2	246	26,254.28	10.42	25.77	114.51	0.652	6	Cell membrane
*sunup08G0020300*	CpSIP2;1	236	25,698.67	9.66	18	114.45	0.672	4	Cell membrane
*sunup06G0004360*	CpXIP1;1	322	34,726.58	8.05	34.7	100.87	0.576	6	Cell membrane
*sunup06G0004370*	CpXIP1;2	309	32,864.93	9.32	26.97	117.02	0.708	6	Cell membrane

**Table 2:** The Ka/Ks ratios of CpAQPs collinearity gene pairs in papaya.

Collinearity Gene Pair	Ka	Ks	Ka/Ks	Evolutionary Selection Model
*CpNIP3;1*–*CpNIP4;1*	0.313639	2.3946	0.130977	Purify selection
*CpTIP1;3*–*CpTIP1;2*	0.134577	2.5987	0.0517862	Purify selection
*CpPIP2;3*–*CpPIP2;2*	0.0862186	1.79504	0.0480314	Purify selection
*CpPIP1;1*–*CpPIP1;4*	0.0699049	2.32253	0.0300986	Purify selection
*CpPIP2;2*–*CpPIP2;1*	0.0955812	1.84551	0.0517912	Purify selection
*CpPIP1;3*–*CpPIP1;2*	0.0493673	1.76285	0.0280042	Purify selection

**Table 3:** Prediction of sequence features of CpAQP proteins in papaya.

Name	NPA Motifs		Ar/R Selectivity Filter				Froger’s Positions					No. of Phosphorylation Site			
	LB	LE	H2	H5	LE1	LE2	P1	P2	P3	P4	P5	S	T	Y	Total
CpPIP1;1	NPA	NPA	F	H	T	R	E	S	A	F	W	15	8	5	26
CpPIP1;2	NPA	NPA	F	H	T	R	Q	S	A	F	W	9	10	6	28
CpPIP1;3	NPA	NPA	F	H	T	R	Q	S	A	F	W	14	10	4	28
CpPIP1;4	NPA	NPA	F	H	T	R	E	S	A	F	W	12	15	7	34
CpPIP2;1	NPA	NPA	F	H	T	R	Q	S	A	F	W	22	9	2	33
CpPIP2;2	NPA	NPA	F	H	T	R	Q	S	A	F	W	16	6	3	27
CpPIP2;3	NPA	NPA	F	H	T	R	Q	S	A	F	W	19	8	4	31
CpPIP2;4	NPA	NPA	F	H	T	R	M	S	A	F	W	26	6	4	36
CpPIP2;5	NPA	NPA	F	H	T	R	M	S	A	F	W	15	7	8	30
CpTIP1;1	NPA	NPA	F	I	A	V	T	S	A	Y	W	23	6	4	33
CpTIP1;2	NPA	NPA	F	I	A	V	T	S	A	Y	W	17	5	1	23
CpTIP1;3	NPA	NPA	F	I	A	V	T	S	A	Y	W	13	4	1	18
CpTIP2;1	NPA	NPA	F	I	G	R	T	S	A	Y	W	10	1	5	16
CpTIP2;2	NPA	NPA	F	I	G	R	T	S	A	Y	W	31	1	2	34
CpTIP3;1	NPA	NPA	F	I	G	R	T	A	A	Y	W	11	8	2	21
CpTIP4;1	NPA	NPA	A	I	A	R	T	S	A	Y	W	10	8	1	19
CpTIP5;1	NPA	NPA	S	F	G	C	T	A	A	Y	W	17	8	1	26
CpNIP1;1	NPA	NPA	M	V	A	R	F	S	A	Y	I	32	7	6	45
CpNIP2;1	NPA	NPA	T	S	G	R	L	S	A	Y	L	35	6	1	42
CpNIP3;1	NPA	NPA	T	V	A	R	F	S	A	Y	V	19	14	3	36
CpNIP4;1	NPA	NPA	M	V	A	R	F	S	A	Y	I	28	7	5	40
CpNIP5;1	NPS	NPV	M	I	G	R	F	T	A	Y	L	18	21	3	42
CpNIP6;1	NPA	NPV	M	I	A	R	F	T	A	Y	L	34	24	1	59
CpNIP7;1	NPA	NPA	V	V	G	R	Y	S	A	Y	M	16	10	2	28
CpSIP1;1	NPT	NPA	F	L	P	I	M	A	A	Y	W	7	4	3	14
CpSIP1;2	NPS	NPA	L	L	P	N	M	A	A	Y	W	13	3	2	18
CpSIP2;1	NPL	NPA	F	L	G	S	I	V	A	Y	W	10	5	/	15
CpXIP1;1	NPV	NPA	F	I	A	K	M	C	A	F	W	39	13	2	54
CpXIP1;2	NPI	NPA	T	T	V	R	V	C	A	F	W	20	19	7	46

## Data Availability

Data is contained within the article and Supplementary Materials.

## Supplementary Materials

The following supporting information can be downloaded at: https://www.mdpi.com/article/10.3390/ijms242417276/s1.
