# Supplementary material for: Genome-Wide Identification and Expression Pattern Profiling of the Aquaporin Gene Family in Papaya (Carica papaya L.)
Source: Int J Mol Sci. 2023 Dec 8;24(24):17276. doi: 10.3390/ijms242417276 (PMC10744249; doi:10.3390/ijms242417276)
Supplement: Supplementary file 1 [file ijms-24-17276-s001.zip › ijms-2706597_Figure S1.pdf]

Figure S1 Results of Multiple sequence alignment

```
                                                    1        10        20
CpPIP11   ........................................MEGKEEDVRLGANKYSERQP
CpPIP12   ........................................MEGKEEDVKLGANKFSERQP
CpPIP13   ........................................MEGKEEDVKLGANKFSERQP
CpPIP14   ........................................MEGKEEDVRLGANRYRERQP
CpPIP21   ........................................MAKDIE....VGGEFQA...
CpPIP22   ........................................MTKDVEV..AEQGEFSA...
CpPIP23   ........................................MTKDAG....EHGSFSA...
CpPIP24   ........................................MSGGEGNEQTVGAEIRG...
CpPIP25   ........................................MSKEVS....EEGQSHG...
CpTIP11   MGSPSAHLIVIFFDPQPVTGYPGSSTSSLSGYKSKSQELRSGDQYLQNHLDPCPYQDTSL
CpTIP12   ............................................................
CpTIP13   ............................................................
CpTIP21   ............................................................
CpTIP22   ............................................................
CpTIP31   ............................................................
CpTIP41   ............................................................
CpTIP51   ............................................................
CpNIP11   .................................MAEISMNSANNNRVVLNVNDGDVGHNS
CpNIP21   ......................................MAGTLTHPNLNNQTDINDLVSV
CpNIP31   ........................................MASKNDSIEEVPPDVEEEGT
CpNIP41   ........................................MSHQDYEVSKVEEGEA....
CpNIP51   ....................................MPESETGTPTASAPATPGTPGGPL
CpNIP61   ....................................MDQKEDVPSAPSTPATPGTPGAPL
CpNIP71   ............................................................
CpSIP11   ............................................................
CpSIP12   ............................................................
CpSIP21   ............................................................
CpXIP11   ......................................MESVIVSSDRSTHSAL......
CpXIP12   ..................................MARNGGAIVVEDEENPYSATRIQPVA
```

```
                  30        40                            50        60
CpPIP11   IGTAAQSQDGGKDYKEPPPAPLFEPGE....................LTSWSFYRAGIAE
CpPIP12   IGTSAQTD...KDYKEPPPAPLFEPGE....................LHSWSFWRAGIAE
CpPIP13   IGTAAQTD...KDYKEPPPAPLFEPGE....................LKSWSFYRAGIAE
CpPIP14   IGTAAQTQD.AKDYTEPPPAPLIEPGE....................LFSWSFYRAGIAE
CpPIP21   ...........KDYHDPPPAPLIDPEE....................LTKWSFYRALTAE
CpPIP22   ...........KDYHDPPPAPLIDVEE....................LTKWSFYRALIAE
CpPIP23   ...........KDYHDPPPAPLFDAVE....................LTKWSFYRALIAE
CpPIP24   ...........RDYEDPPPASLIDMEE....................LKKWSFYRAVIAE
CpPIP25   ...........KDYVDPPPAPLIDLAE....................IKLWSFYRALIAE
CpTIP11   PLTHPSFHLNQTNMPIRNIAIG.RPEE....................ATHPDALKAALAE
CpTIP12   .............MPISQIAIG.SPAE....................IARPDALKAALAE
CpTIP13   .............MPIYRVAIG.APRE....................LSHPSAIRAALAE
CpTIP21   ...............MACIAFG.RFDD....................IYSLASLKAYLAE
CpTIP22   ...............MVKIALG.SFGD....................SFSVGSLKAYLSE
CpTIP31   .............MPVRRYAFG.RVEE....................VTHPDSIRATLAE
CpTIP41   ...............MAKIALG.TRHE....................VTKPDCIRALVVE
CpTIP51   ...............MAPASLTSRFGQ....................SITKNAFRSYLAE
CpNIP11   LPASTSESLE.....KRDSAL..........................SFSVPFIQKLMAE
CpNIP21   ESPISDRSSI.....WKSFEH..........................HYPPCFLRKVAAE
CpNIP31   ATSTNRLEPT.....SKNSSN..........................LWAFTLAQKVIAE
CpNIP41   ..CSRSSSRI.....PESSST..........................SWVVELLQKVIAE
CpNIP51   ISGLRVDSLS...YDRKSMAR.CKCLPVTAPTWGQP...HTCFIDFPAPDVSLTRKLGAE
CpNIP61   FGGFRRERSSSGFFRSKSLINTCNCFTLRDWSLEDPSVLPPVTCTLPHPPVSLARKVGAE
CpNIP71   ............................................................
CpSIP11   ..................................................MGPIKAAIGD
CpSIP12   ..................................................MGVIRSAIGD
CpSIP21   ..................................................MAKIRLLISD
CpXIP11   STSIENYDKTSPTRISKNNSFLASIG..................AHEFFSQEMWGAAITE
CpXIP12   STPMAQQRNTEKGKKKTPTTLTGVLG..................FKDLFSLKVWRASLAE
```

```
                  70        80               90       100       110
CpPIP11   FMATFLFLYITVLTVMGVVKESS.......KCSTVGIQGIAWAFGGMIFALVYCTAGISG
CpPIP12   FMATFLFLYITVLTVMGVNRAPN.......KCASVGIQGIAWAFGGMIFALVYCTAGISG
CpPIP13   FIATFLFLYITILTVMGVKRSDS.......MCASVGIQGIAWAFGGMIFALVYCTAGISG
CpPIP14   FVATFLFLYITVLTVMGVVKSPT.......KCSTVGIQGIAWAFGGMIFALVYCTAGISG
CpPIP21   FVATLLFLYITVLTVIGYKSQTD.KNQGGDDRGGVGILGIAWAFGGMIFILVYCTAGISG
CpPIP22   FVATLLFLYVTVLTVIGHKSQTDLTKAGTDVCGGVGLLGIAWAFGGMIFILVYCTAGISG
CpPIP23   FIATLLFLYVTVLTVIGHKSQH.....AADQCGGVGILGIAWAFGGMIFILVYCTAGISG
CpPIP24   FVATFLFLYVGIATVIGNKKQIH.......PCDGVGLLGISWSFGGMIFILVYCTAGISG
CpPIP25   FIATLLFLYVTIATVIGHKKQAG.......PCDGVGILGIAWAFGGMIFILVYCTAGISG
CpTIP11   FISMLIFVFAGEGSGMAFNKLTNNG.....AATPAGLIAAAIAHAFALFVAVAVGANISG
CpTIP12   FISMLIFVFAGEGSGMAFNKLTNYG.....SATPAGLISASLAHAFALFVAVSVGANISG
CpTIP13   FFSMVIFVFAGEGSGMAFDKLTNNG.....SSTPAGLVAASLAHAFALFVAVSVGANISG
CpTIP21   FISTLLFVFAGVGSAMAFNKLTSDA.....PLDPSGLVAIAVCHGFALFVAVAVAANISG
CpTIP22   FIATLLFVFAGVGSAIAFGKVTSDG.....ALDPAGLVAIAVAHAFALFVGVAIAANISG
CpTIP31   FLSTLVFVFAGEGSVLALDKLYRETG.GDVSRDPSGLVLIALAHALSLFSAVSASINISG
CpTIP41   FITTFLFVFTGVGSAMAADRLVGNG........LLGLFAVAVAHALVVAVMISAG.HISG
CpTIP51   FISTFFYVLTVVGSSMSARKLMGSG.....AEDPSGLVIVAIANSLGLSYTVYVASNISG
CpNIP11   FFGTFFLIFAGCASVAVNDEYEK..........VVTLPGISIVWGLAVMVLVYSVGHISG
CpNIP21   VIATYLLVFVTCGSAAISAIDES..........RVSKLAASVAGGLIVTVMIYAVGHISG
CpNIP31   LVGTYFIIFSGCGAVAVNKIYG...........SVTFPGICVTWGLIVTVMIYTVGHVSG
CpNIP41   VLGTYFVMFSGCGSVVVNKIYG...........SVTFPGICVVWGLIVMVMVYSVGHISG
CpNIP51   FVGTFILIFAATAAPIVNQKYNG..........AETLIGNAACSGLAVMIIILSTGHISG
CpNIP61   FIGTLILIFAGTATAIVNQKSQG..........SETLIGLAASTGLAVMIVILSTGHISG
CpNIP71   MVGTFILVLCVCGIIASQQLMRG..........EVGLMEYAATAGLTVVVVIFCIGPISG
CpSIP11   MILTFMWNFFASTFGLMT..VMILGAAGLQGVAWAPVLITTLLVFIFVFIFGLIGDALGG
CpSIP12   SVLTSMWVFNLPVIGLLAGRASDFLRTHYISLPFTGLFITILLATINVLLFTLLGTLLGG
CpSIP21   FIVSFMWVWSGVLIKIFVHRVLGLGHEPRAEIIRG......AMAIVNMFFFAFLGKVGKG
CpXIP11   LVGTTCLLFTLTISIVACLNFHE..........VESKLLVPIVVFVILFFFLMATIPISG
CpXIP12   LLGTAVLVFAMDTIVISSYETQT..........KTPHLIMSFLVAITVTILLLATSPISG
```

```
             120       130       140       150       160
CpPIP11  GHINPAVTFGLLLARKLS....LTRAIFYMVMQCLGAICGAGVVKGFEGSATFELKGGGA
CpPIP12  GHINPAVTFGLFLARKLS....LTRSIFYMVMQCLGAICGAGVVKGFQ.PRPYQMLGGGA
CpPIP13  GHINPAVTFGLLLARKLS....LTRAVFYMIMQCLGAICGAGVVKGFQ.PGPYQRLGGGA
CpPIP14  GHINPAVTFGLLLARKLS....LVRAVFYMIMQCLGAICGAGVVKAFE.KTQYEMLGGGA
CpPIP21  GHINPAVTFGLFLARKVS....LVRAVLYMVAQCLGAICGCGLVKAFQ.KACYNRYGGGA
CpPIP22  GHINPAVTLGLFLARKVS....LIRAIMYMVAQCLGAICGVGLVKAFQ.SSFYNRYGGGA
CpPIP23  GHINPAVTFGLFLARKVS....LVRAVMYMVAQCLGAICGVGLVKAFQ.KSFYNRFGGGA
CpPIP24  GHINPAVTLGLFAARKMS....LIRAAAYMVSQCGGAICGVGLVKLFM.TRSYNMHGGGA
CpPIP25  GHINPAVTFGLFLARKVS....LIRAVAYMVAQCLGAICGVGLVKAFM.KNYYNRLGGGA
CpTIP11  GHVNPAVTFGAFVGGNIS....LLRGILYWIAQLLGSVAACALLKFATGGLTTSAFA...
CpTIP12  GHVNPAVTFGAFVGGHIS....LFRSVLYWIAQCLGSVLACLLLKFSTGGLETSAFA...
CpTIP13  GHVNPAVTFGAFLGGNIT....FFRSILYWIAQLLGSVVACLLLKFATGNMETAAFG...
CpTIP21  GHVNPAVTLGLAVGGQIT....ILTGIFYWIAQLLGSIVACFLLKAVTGCMAIPIHS...
CpTIP22  GHLNPAVTLGLAIGGNIT....LLTGLFYWIAQCLGSIVACGLLKFVT.DLSVPTHS...
CpTIP31  GHVNPAVTFGALLGGRIS....VLRAFYYWLAQLLGAIVACLLLRLVTAGMRPVGFR...
CpTIP41  GHLNPAVTLGLLFGGHIT....LVRSILYWIDQLLASSIACILLKYLTGGLDTPIHT...
CpTIP51  GHVNPAVTFARAVGGHVS....VPTALFYWVSQMLASVMASLILRVMTVAQHVPTYA...
CpNIP11  AHFNPAVTIAFASCRRFP....LKQVPAYILVQLLGSTLAAGTLRLLFNGQHQVFTGTAP
CpNIP21  AHMNPAVTLAFAALRHFP....WKQVPFYAAAQVTGAISAAFTLRVLLHP.IKLIGTTSP
CpNIP31  AHFNPAVTIAFTIFQKFP....PSEVLFYIVAQFLGSILASGTLALMFDITPNAYFGTTP
CpNIP41  AHFNPAVTITFAIFRRFP....FKQVPLYILAQLLGAILASYTLWIIFHVNEESFFGTVP
CpNIP51  AHLNPSVTICFAALRHFP....WAQVPAYIAAQISASICASFTLKGVFHPFLSG.GVTVP
CpNIP61  AHLNPAVTISFAALHHFP....WKHVPAYIAAQTVGSLCAAFALKIVFHPMMGG.GVTVP
CpNIP71  AHVNPAVTIAFAIFGHFS....WSRVPFYILAQMLGSTLATWAGRSVYGVRADLMATRPV
CpSIP11  ASFNPTGTAAFYAAGVG..SDSLLSMAIRFPAQAVGAVGGALAITEVMPIQYKHMIGGPS
CpSIP12  ASFNPSTTVSFHAAGLTKPGSSLISMAVRLPAQAAGGAIGAMGIWQVMPVG..WLKGGPS
CpSIP21  ASYNPLTVLAPAVSGDF..SSFLFSLGCRIPVQVIGSIVGVRFILETIPEAG....LGPR
CpXIP11  GHMNPVFTFIATLKGIIT....ITRAAFYFLAQCLGSIISFIIIKSVMNHDSATKFSLGG
CpXIP12  GHINPIVTVAAVLTGLIS....VSRAIVYILAQCIGGILGALALKAVVNSTIQQTFSLGG
```

```
         170                   180       190       200         210
CpPIP11  N...........VVNHGYTKGDGLGAEIVGTFVLVYTVLSATDAKRNARDS...HVPILA
CpPIP12  N...........MVNHGYTKGDGLGAEIVGTFVLVYTVFSATDAKRNARDS...HVPILA
CpPIP13  N...........VVNHGYTKGDGLGAEIVGTFVLVYTVFSATDAKRNARDS...HVPILA
CpPIP14  N...........TVGPAYSKTAGLGAEIVGTFVLVYTVFSATDAKRNARDS...HVPILA
CpPIP21  N...........QLALGYSTGTGLGAEIIGTFVLVYTVFSATDPKRNARDS...HVPVLA
CpPIP22  N...........SLNGGYNKGTGLGAEIIGTFVLVYTVFAATDPKRNARDS...HVPVLA
CpPIP23  N...........TLADGYNVGTGLGAEIIGTFVLVYTVFSATDPKRSARDS...HVPVLA
CpPIP24  N...........SVAPGFSTTTGLGAEIIGSFVLVYTVFSATDPKRSARES...FIPVLA
CpPIP25  N...........TVATGYNTGTALGAEIIGTFVLVYTVFSATDPKRSARDS...HVPVLA
CpTIP11  .............LSSGVGVWNAFVFEIVMTFGLVYTVYATAVDPKKG......SLGTIA
CpTIP12  .............LSSGVGELNALVFEIVMTFGLVYTVYATAIDPKRG......NIGIIA
CpTIP13  .............LSSGVSPMNALVFEIVMTFGLVYTVYATAVDPKKG......NLGTIA
CpTIP21  .............VAAEVGVIGGLVMEIIITFALVYTVYATAADPRKG......SLGTIA
CpTIP22  .............VGSGMSVLEGVVMEIVITFALVYTVYATAADPKRG......SLGIIA
CpTIP31  .............VASGVGELNGLVLEMVLTFGLMYTVYATTIDPKRG......SIGIIG
CpTIP41  .............LASGVGYGQGVVWEIVLTFSLLFTVYATIVDPKKG......SLDGLG
CpTIP51  .............IAEQMTGFGASVLEGVLTFALVYTIYAAG.DPRRG......QMGAIG
CpNIP11  ..............SGSDM..QSFGIEFIITFYLMFIISGVATDNR........AIGELA
CpNIP21  ..............AGSDI..QALIMEIVVTFSMMFITSAVATDTK........AVGELA
CpNIP31  ..............VGSNG..QSLAIEIIITFLLTFVIFGASIDER........AIGQLG
CpNIP41  ..............VGSDL..QSLWIEIIISFILMFVISGVATDNR........AIGELA
CpNIP51  ..............SVSLG..QAFALEFLISFILLFVIT...............AVGELA
CpNIP61  ..............SPSVGYAQAFALEFIISFNLMFVVTAVATDTR........AVGELA
CpNIP71  ..............QGCFA...AFWVEFFGTFIIMFLSAALICEAH........TIGHLS
CpSIP11  ...............LKVELHTGAVAEGILTFLITFAVLVIILKGPRN.........SVL
CpSIP12  ...............LKVDWHTGALAEGLLAFAHCLSVLVVVRGPRS..........VFV
CpSIP21  ...............LKVDIHQGALTEGFLTFAIVMISLGLAAKIPGS.........FFM
CpXIP11  CSIKGHGS.......TGLHLGVALMLEFSCTFLVLFVAVNVAFDKRRSKELGVSKVCAQI
CpXIP12  CTLTVVVPGRHGPVVIGLETGQALWLEIICTFVFLFASIWVAFDYRQAKALGRFMVCLVI
```

```
            220       230       240       250       260       270
CpPIP11  PLPIGFAVFLVHLATIP..ITGTGINPARSLGAAIIFNRDKAWDDHWIFWVGPFIGAALA
CpPIP12  PLPIGFAVFLVHLATIP..ITGTGINPARSLGAAIIYNDDTAWDHHWVFWVGPFIGAALA
CpPIP13  PLPIGFAVFLVHLATIP..ITGTGINPARSLGAAIIFNTDHAWDDHWIFWVGPFIGAALA
CpPIP14  PLPIGFAVFLVHLATIP..VTGTGINPARSLGAALIYNKSQAWDDHWIFWVGPFIGAALA
CpPIP21  PLPIGFAVFMVHLATIP..VTGTGINPARSFGAAVIFNQDKPWDDHWIFWVGPFIGAAIA
CpPIP22  PLPIGFAVFMVHLATIP..ITGTGINPARSFGAAVIFNDEKAWDDHWIFWVGPFIGAAIA
CpPIP23  PLPIGFAVFMVHLATIP..VTGTGINPARSFGAAVIFNDKKAWDDHWIFWVGPFIGAAIA
CpPIP24  PLPIGLAVFMVHLATIP..ITGTGINPARSLGAAVVYNNQQVWDEQWIFWVGPFLGALAA
CpPIP25  PLPIGFAVFMVHLATIP..ITGTGINPARSFGAAVIWNNKKGWDDHWIFWVGPFVGALAA
CpTIP11  PIAIGFIVGANILAGGA..FDGASMNPAVSFGPAVVSWS...WDNHWVYWAGPLIGGGLA
CpTIP12  PIAIGFIVGANILAGGA..FDGASMNPAVSFGPAVVSWT...WNSHWVYWVGPFAGAGIA
CpTIP13  PIAIGFIVGANILAGGA..FDGASMNPAVSFGPAVVSWT...WTHHWVYWVGPFIGAAIA
CpTIP21  PIAIGFIVGANILAAGP..FSGGSMNPARSFGPAVASGN...FCGIWIYWVGPLVGGGLA
CpTIP22  PIAIGFIVGANILAAGP..FSGGSMNPARSFGPAVVSGD...FTDNWVYWLGPLVGGALA
CpTIP31  PLAIGLIVGGNILVGGP..FDGGSMNPARAFGPALVGWR...WRNHWIYWVGPFVGGGLA
CpTIP41  PMLTGFVVGANILAGGA..FSGASMNPARSFGPALVSWD...WTDHWVYWVGPLMGGGLA
CpTIP51  PLVIGMAAGANFLAAGP..FSGGSMNPACAFGSAVVAGS...FKNQAVYWVGPLIGATIG
CpNIP11  GLAVGATVLLNVMFAGP..ISGASMNPARSLGPAIVFSR...YKGIWIYIFSPVLGAISG
CpNIP21  GIAVGSAVCITSILAGP..VSGGSMNPARSIGPALASQY...YKGIWVYLVGPVTGTLLG
CpNIP31  GIAVGMTVMLNVFVAGP..ISGASMNPARSLGPAFVKHE...FKGLWIYVIGPVAGAIAG
CpNIP41  GIAVGMTIILNVFVAGP..VSGASMNPARSIAPAIVMHV...YKGLWVYIVGPPIGTILG
CpNIP51  GIAVGATVMLNILVAGP..SSGGSMNPVRTLGPAVAAGN...YKAIWVYLLAPILGGLVG
CpNIP61  GIAVGATVMLNILIAGP..STGASMNPVRTLGPAIAANN...YKAIWVYLIAPILGALSG
CpNIP71  GFVVGIAIGLAVLITGP..VSGGSMNPARSLGPAIVSWD...FDNIWIYMIAPVVGAIAG
CpSIP11  KTWLLAVATVALVLSGS.AYTGPAMNPAIAFGWAYQNNWHNSWEHFYVYWICPFIGAIFA
CpSIP12  KVLLLAMVTTGLVRVGS.GYTGPSLNPANAFGWAYVKNWHNSLELYYVYWVGPLVGATMA
CpSIP21  KTWISSVSKLALHILGS.DLTGGCMNPASVMGWAFARGDHITKEHIFVYWLAPVEATLLA
CpXIP11  AGAMALAVFVSITVTGQTAYAGAGLNPAKCFGAAILQGG.LLWKGHWVFWVGSFFACIVY
CpXIP12  GVVVGLIVFVSTTVTATKGYAGVGMNPARCLGPALIRGG.HLWSGHWVFWAGPVIACVAF
```

```
                  280
                    .
CpPIP11   ALYQQVVIRAIPFKSK..............................
CpPIP12   ALYHQIVIRAIPFKTRG.............................
CpPIP13   AVYHQIVIRAIPFKTRA.............................
CpPIP14   ALYHQIVIRAIPFRSK..............................
CpPIP21   AFYHQFVLRASGSKSLGS.....LRSSSNI................
CpPIP22   AIYHQYVLRAAAIKALGS.....FRSNA..................
CpPIP23   AFYHQFILRAAAVKALGS.....FRSQSHV................
CpPIP24   AAYHEYVLRAAAVKALLS.....FRPH...................
CpPIP25   AAYHQYILRAAAIKALGS.....FRSNPTN................
CpTIP11   GLIYDFFFI.SHSH.......EQLPTADY.................
CpTIP12   ALVYEIIFIGSSTH.......EQLASADF.................
CpTIP13   AIVYDNIFIGNDSH.......EPLPTNDF.................
CpTIP21   GLIYGNIFISS.SHQS........LPSDC.................
CpTIP22   GLVYGDIFIGSYSPVS........ASQDYP................
CpTIP31   ALVYEFMVIPSTTEPPLITGHQPLAPEDY.................
CpTIP41   GFMYENFFIVRSHVLVS.......HQDDSC................
CpTIP51   GLLYDNVVFPPEAIDSLTG....ISERPVV................
CpNIP11   AWVYNMVRYTDKPLREI.TKSSSFLKSARSCST.............
CpNIP21   AYSYNLIRVKDEPVQAISPRSFSFKLRRMKSHEEQINMKDPLNSL.
CpNIP31   ASAYSLVRAGDRP.....SETLSFLTGSSK................
CpNIP41   GAAYNLIRFTDKPLREI.TRTGSFLKSISRRN..............
CpNIP51   AGTYTAVKLRDDEAE.....PPRQVRSFRR................
CpNIP61   AGIYTAVKLPEEDADTH..EKPSTARSFRR................
CpNIP71   VLLYQFLRLKHRPCTAT..SSPSTVSYLVTP...............
CpSIP11   ALVFRIIFPPTEVKKKK.......QKKA..................
CpSIP12   AWVFRVLFAPSLVKKKK.......KKKKRE................
CpSIP21   VWTFKLVTKSLTEDKAK.......LKAKSE................
CpXIP11   YGFSLTLPKQGLDWVEGEYDAMRLAKACWGTKDFPNSSLQEKGDGP
CpXIP12   ALYIKMIPREHFHGGD..............................
```
