# Supplementary material for: Genome-Wide Identification and Expression Pattern Profiling of the Aquaporin Gene Family in Papaya (Carica papaya L.)
Source: Int J Mol Sci. 2023 Dec 8;24(24):17276. doi: 10.3390/ijms242417276 (PMC10744249; doi:10.3390/ijms242417276)
Supplement: Supplementary file 1 [file ijms-24-17276-s001.zip › ijms-2706597_Figure S2.pdf]

Figure S2 Diagram of predicted phosphorylation sites of papaya AQPs by NetPhos 3.1

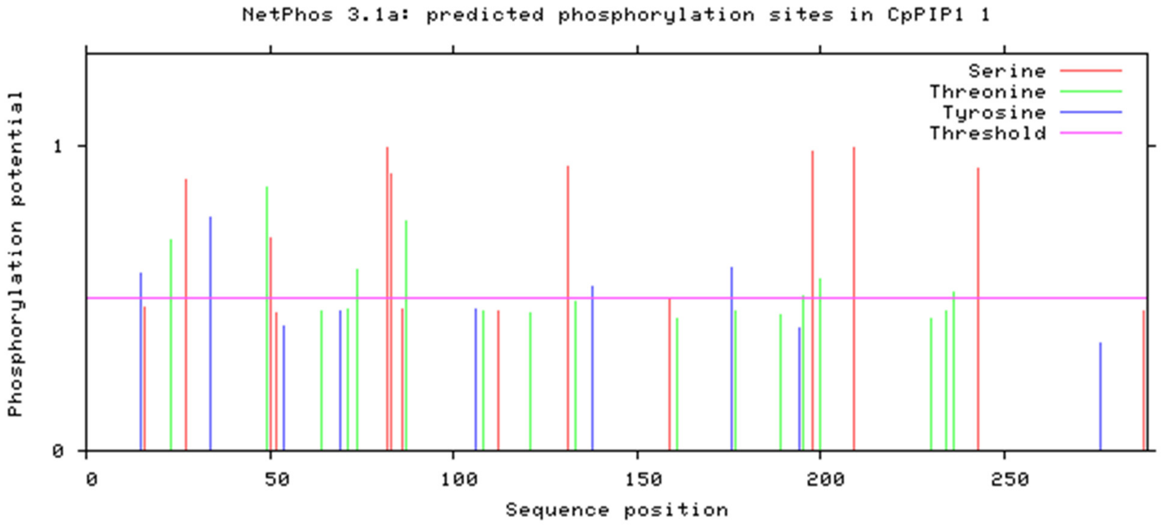

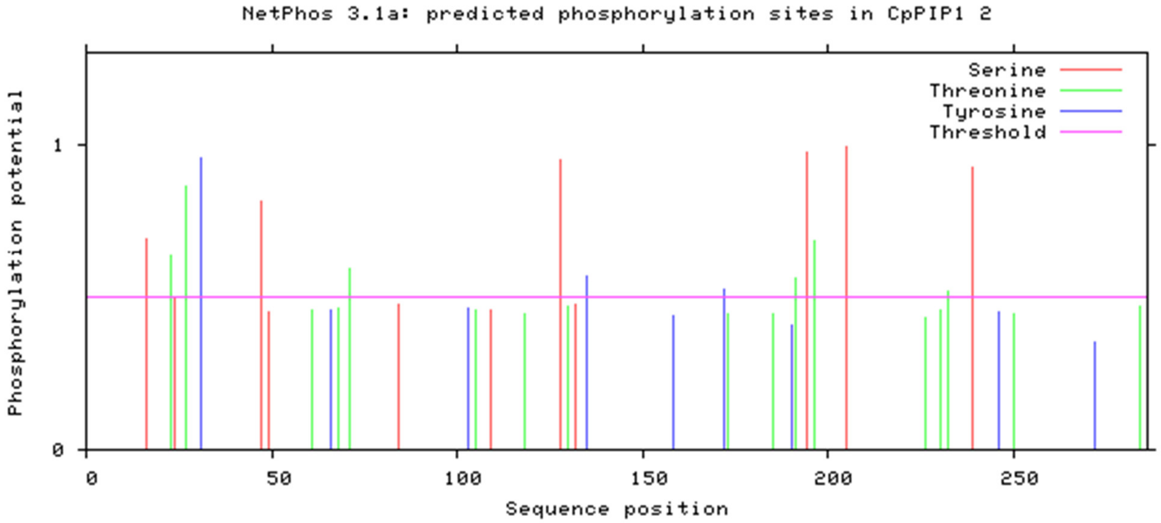

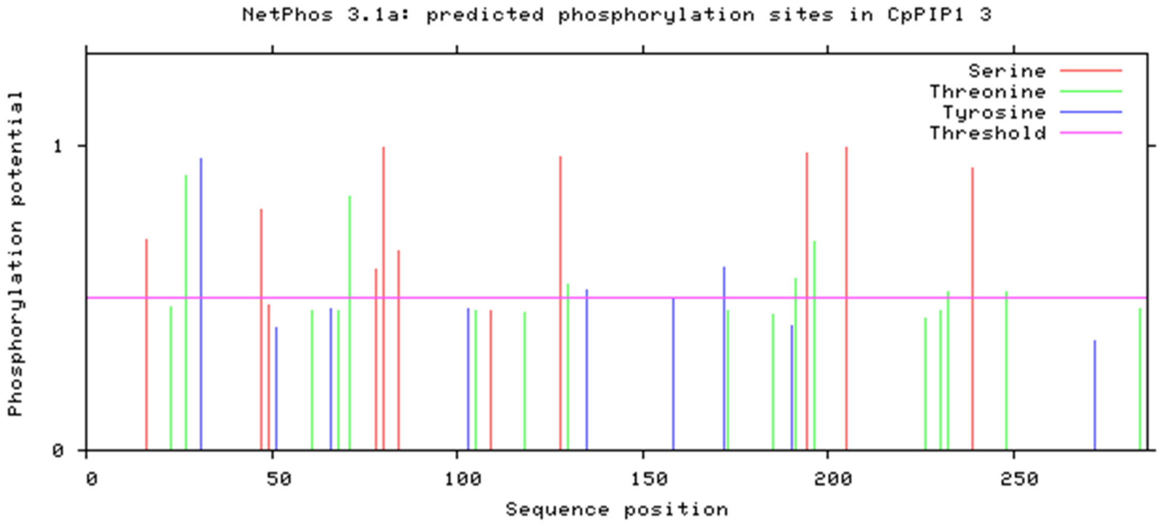

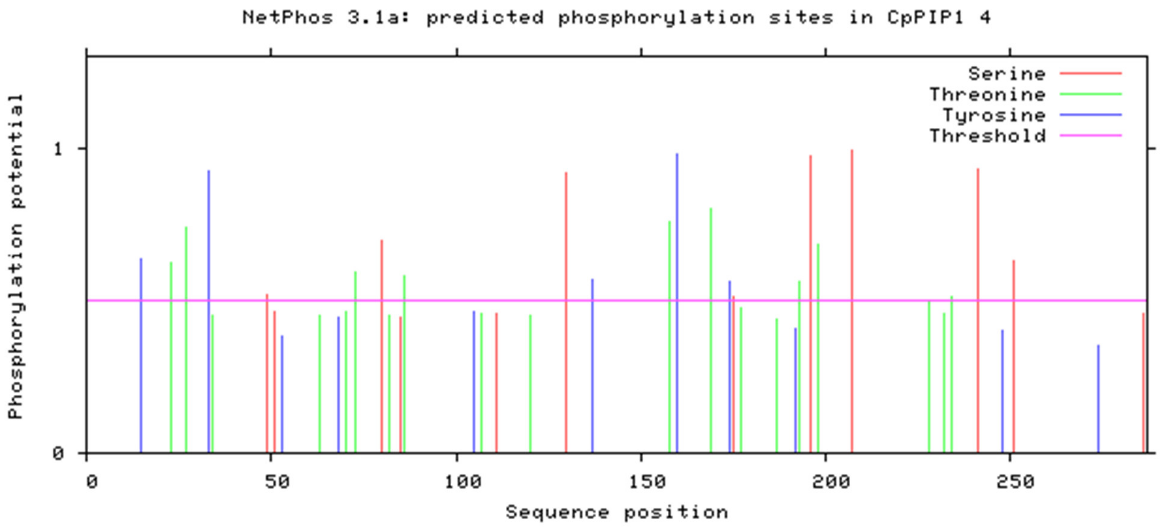
NetPhos 3.1a: predicted phosphorylation sites in CpPIP1 4
Serine
Threonine
Tyrosine
Threshold
Phosphorylation potential
1
0
0
50
100
150
200
250
Sequence position

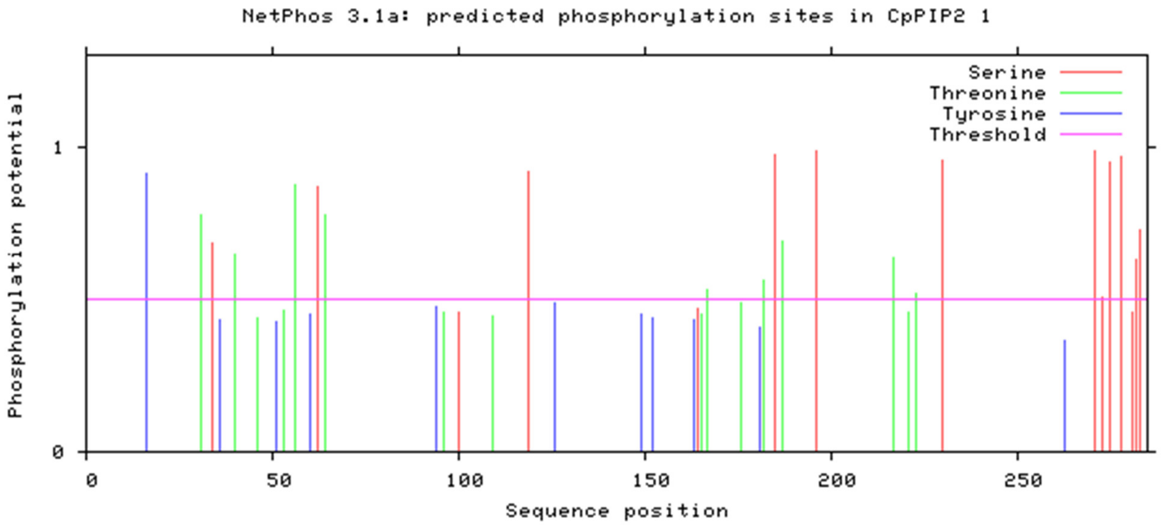
NetPhos 3.1a: predicted phosphorylation sites in CpPIP2 1
Serine
Threonine
Tyrosine
Threshold
Phosphorylation potential
1
0
0
50
100
150
200
250
Sequence position

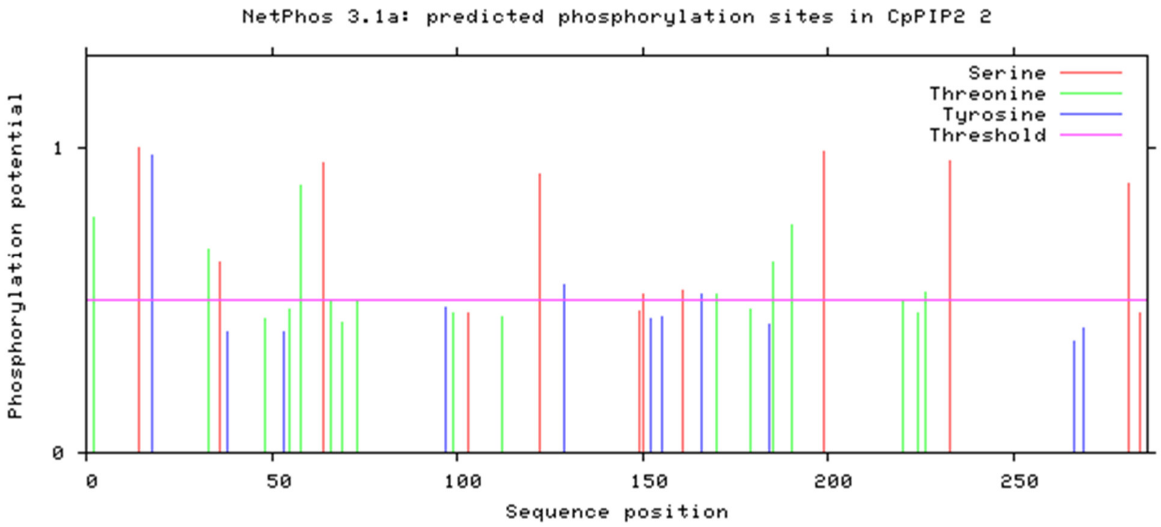
NetPhos 3.1a: predicted phosphorylation sites in CpPIP2 2
Serine
Threonine
Tyrosine
Threshold
Phosphorylation potential
1
0
0
50
100
150
200
250
Sequence position

NetPhos 3.1a: predicted phosphorylation sites in CpPIP2 3

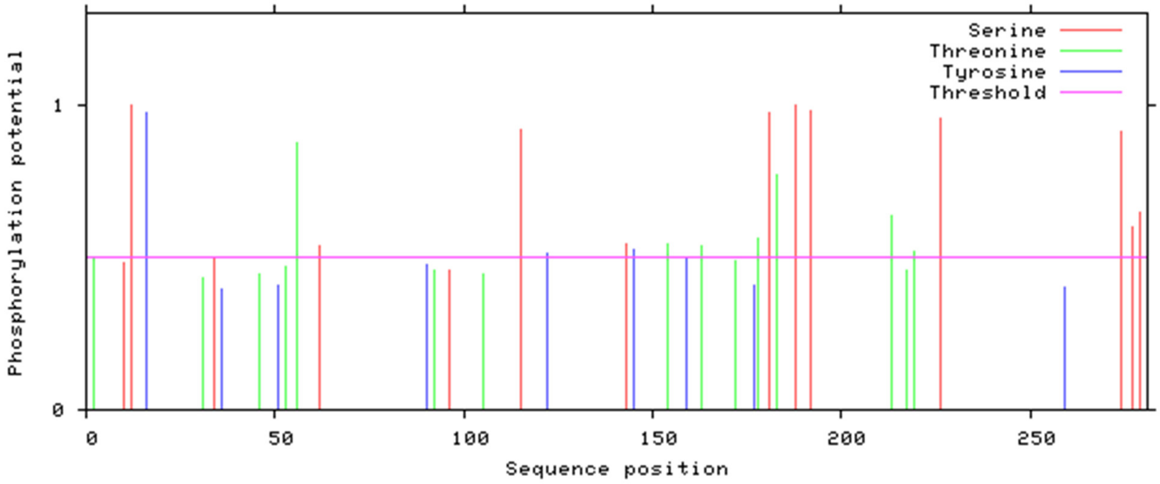

NetPhos 3.1a: predicted phosphorylation sites in CpPIP2 4

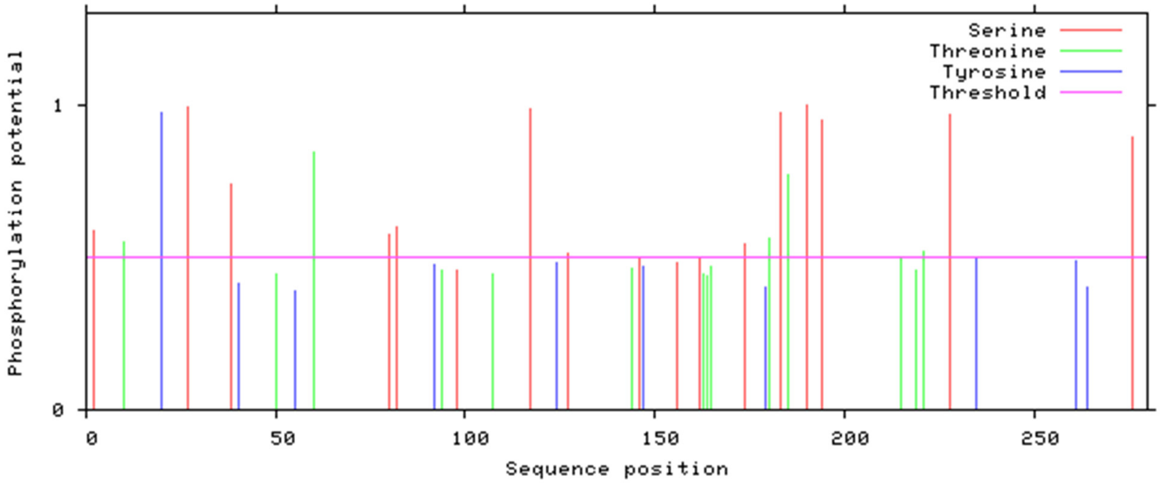

NetPhos 3.1a: predicted phosphorylation sites in CpPIP2 5

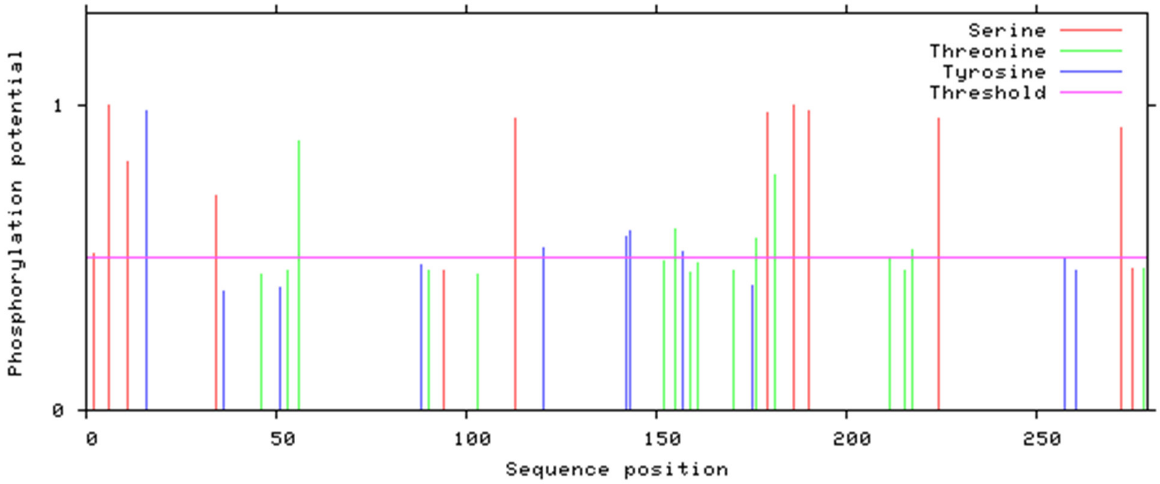

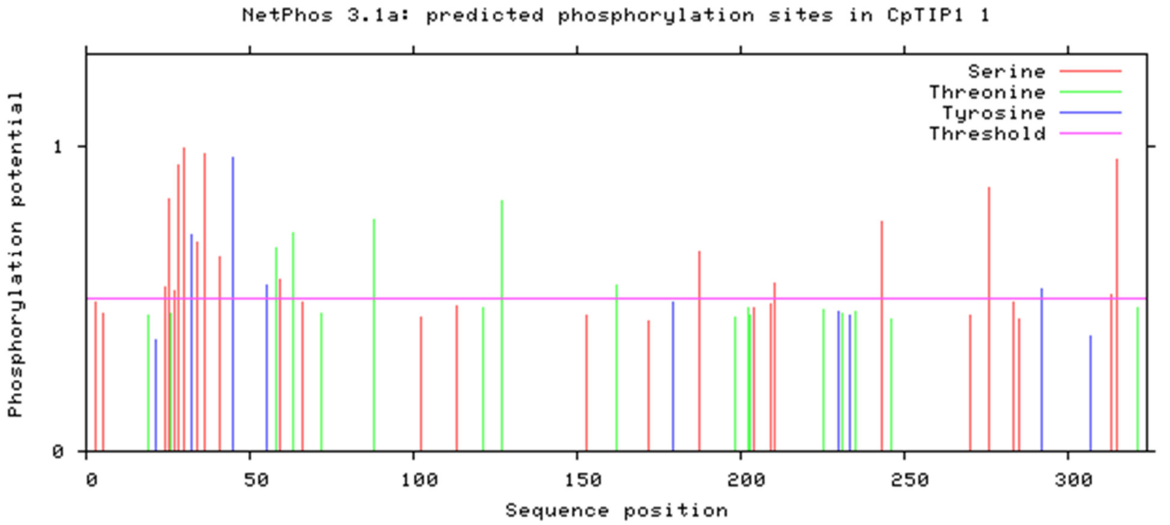
NetPhos 3.1a: predicted phosphorylation sites in CpTIP1 1
Serine
Threonine
Tyrosine
Threshold
Phosphorylation potential
Sequence position

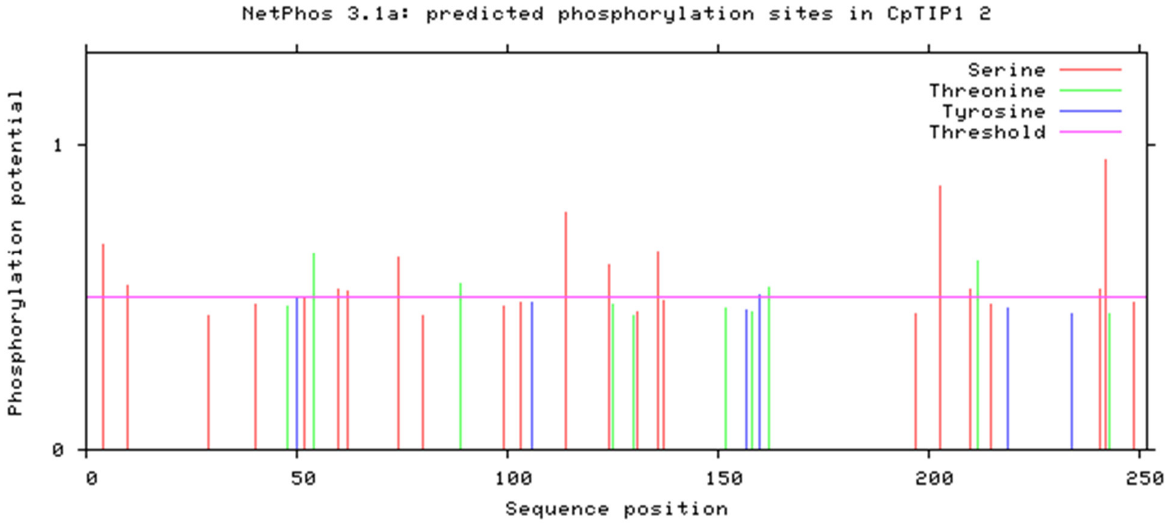
NetPhos 3.1a: predicted phosphorylation sites in CpTIP1 2
Serine
Threonine
Tyrosine
Threshold
Phosphorylation potential
Sequence position

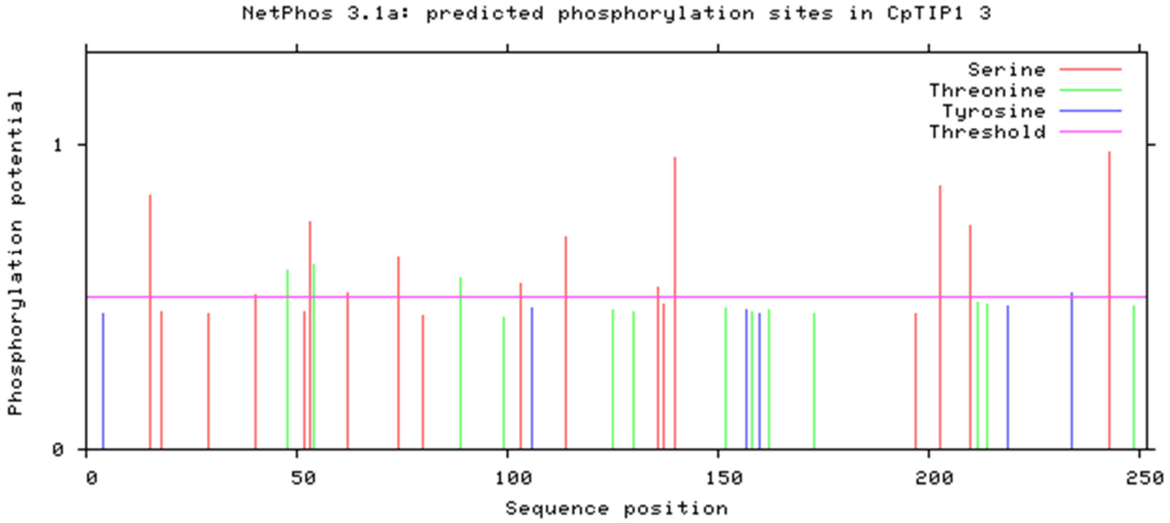
NetPhos 3.1a: predicted phosphorylation sites in CpTIP1 3
Serine
Threonine
Tyrosine
Threshold
Phosphorylation potential
Sequence position

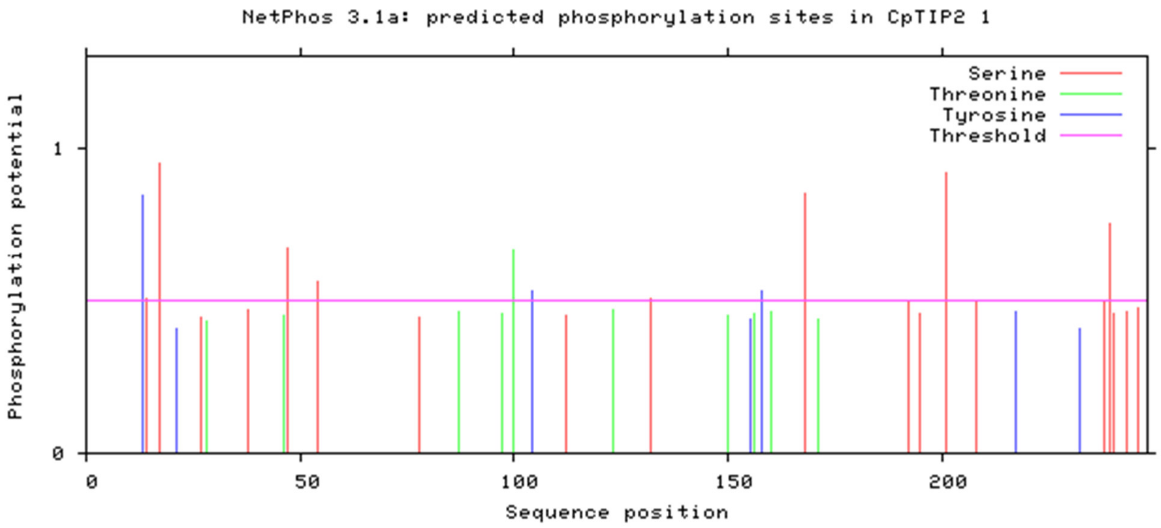
NetPhos 3.1a: predicted phosphorylation sites in CpTIP2 1
Serine
Threonine
Tyrosine
Threshold
Phosphorylation potential
Sequence position
0
1
50
100
150
200

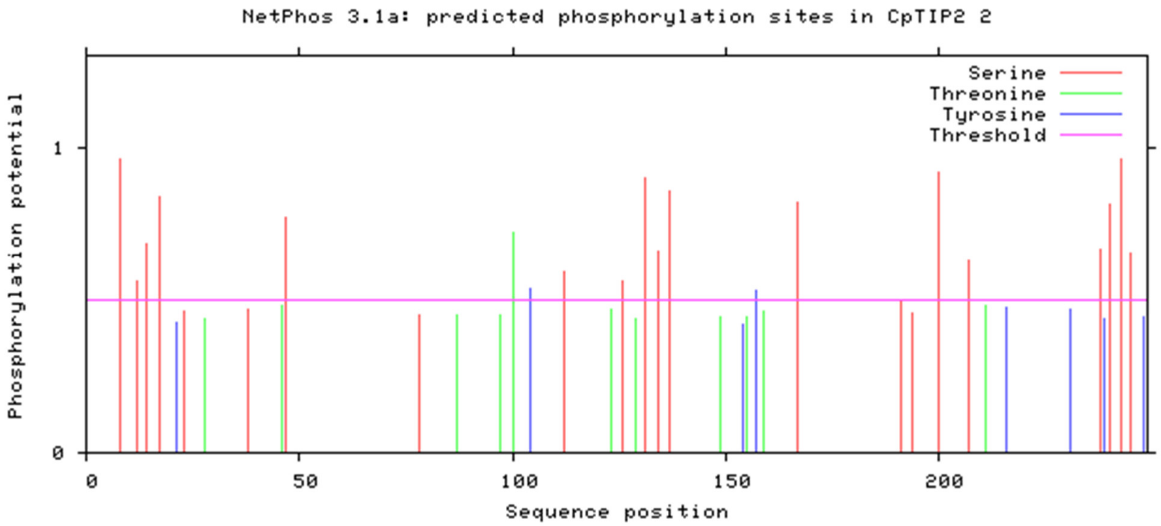
NetPhos 3.1a: predicted phosphorylation sites in CpTIP2 2
Serine
Threonine
Tyrosine
Threshold
Phosphorylation potential
Sequence position
0
1
50
100
150
200

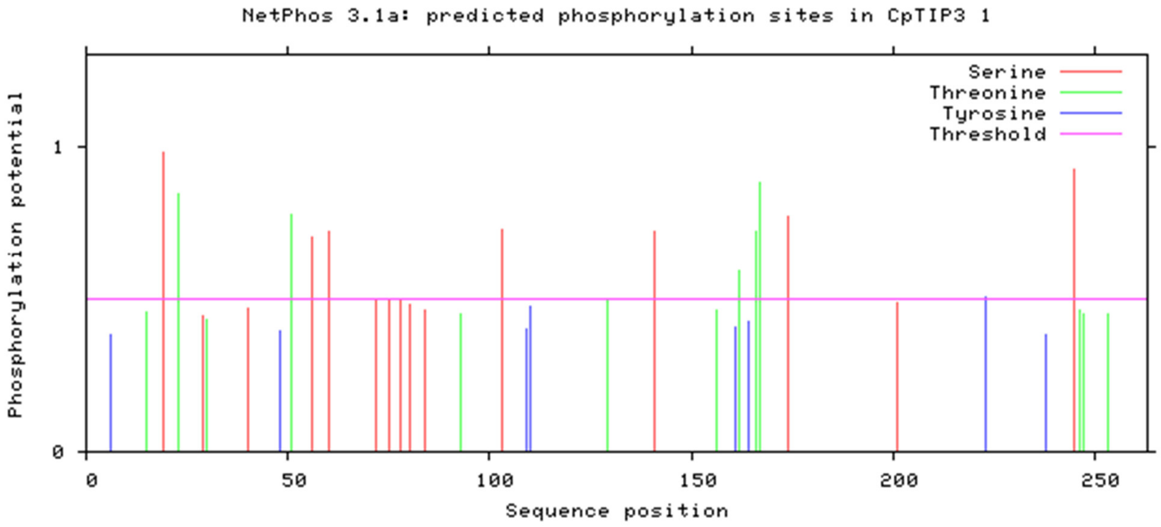
NetPhos 3.1a: predicted phosphorylation sites in CpTIP3 1
Serine
Threonine
Tyrosine
Threshold
Phosphorylation potential
Sequence position
0
1
50
100
150
200
250

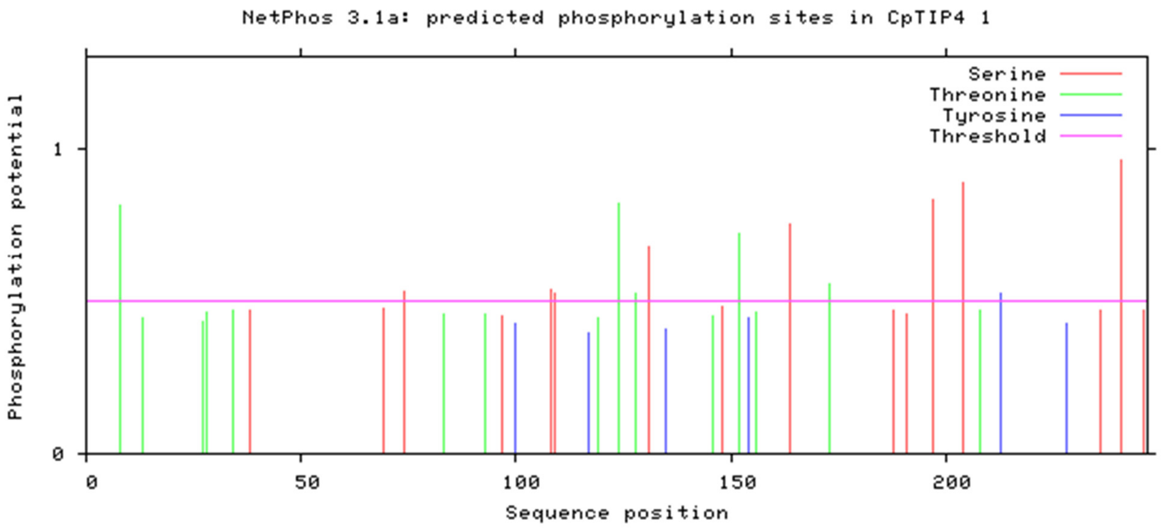
NetPhos 3.1a: predicted phosphorylation sites in CpTIP4 1
Serine
Threonine
Tyrosine
Threshold
Phosphorylation potential
Sequence position
0
1
0
50
100
150
200

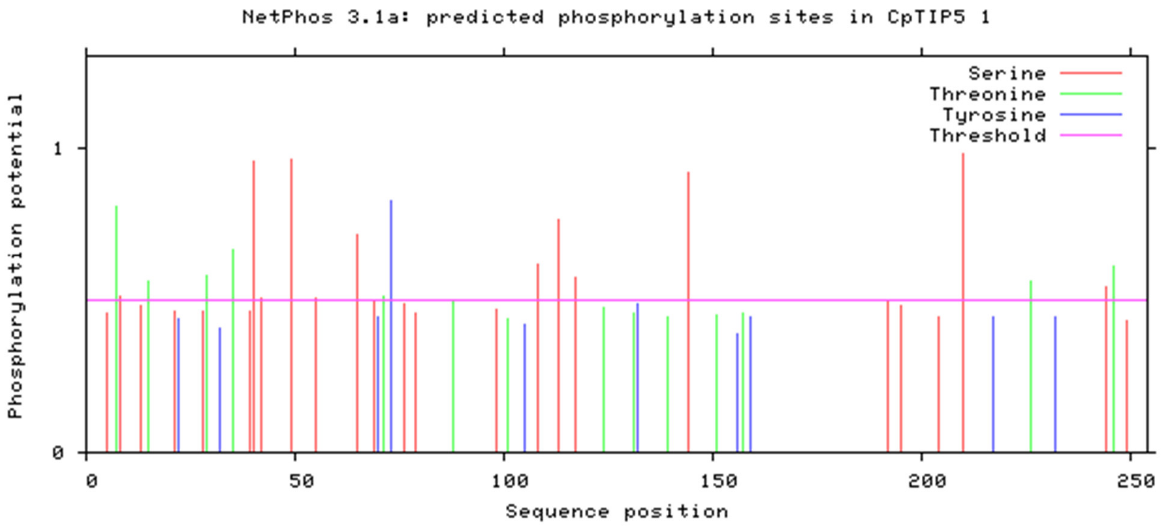
NetPhos 3.1a: predicted phosphorylation sites in CpTIP5 1
Serine
Threonine
Tyrosine
Threshold
Phosphorylation potential
Sequence position
0
1
0
50
100
150
200
250

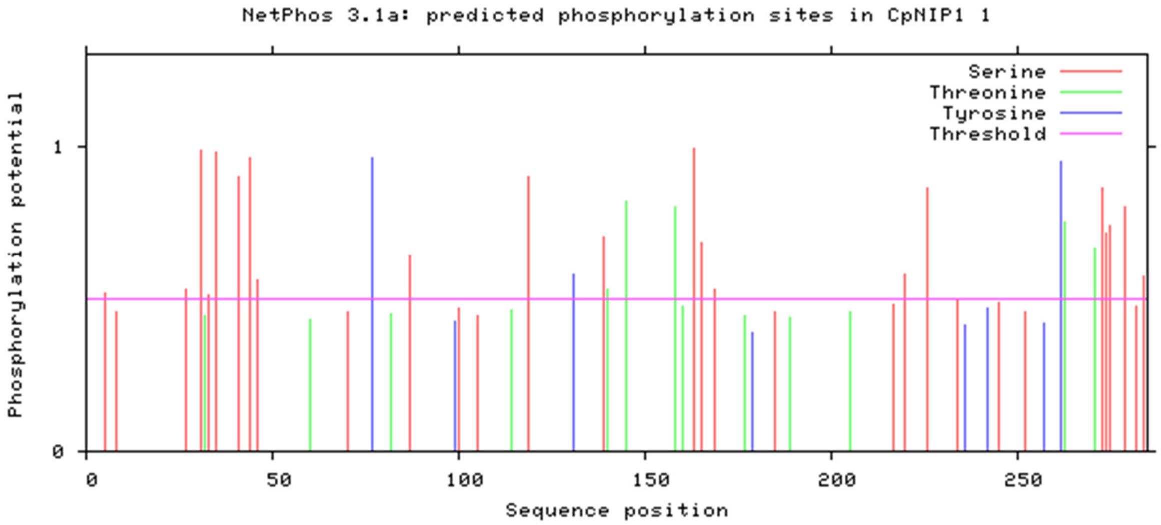
NetPhos 3.1a: predicted phosphorylation sites in CpNIP1 1
Serine
Threonine
Tyrosine
Threshold
Phosphorylation potential
Sequence position
0
1
0
50
100
150
200
250

NetPhos 3.1a: predicted phosphorylation sites in CpNIP2 1

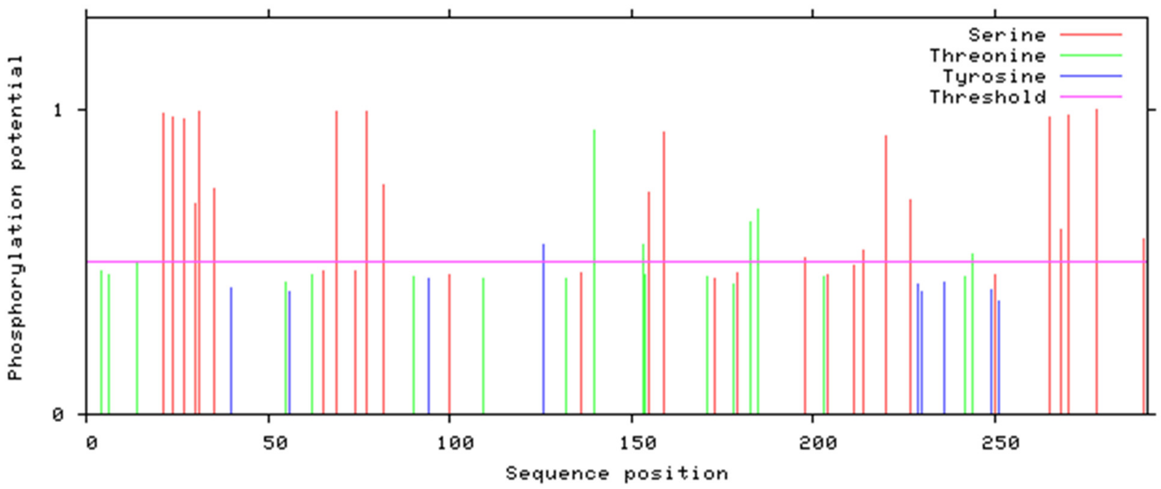

NetPhos 3.1a: predicted phosphorylation sites in CpNIP3 1

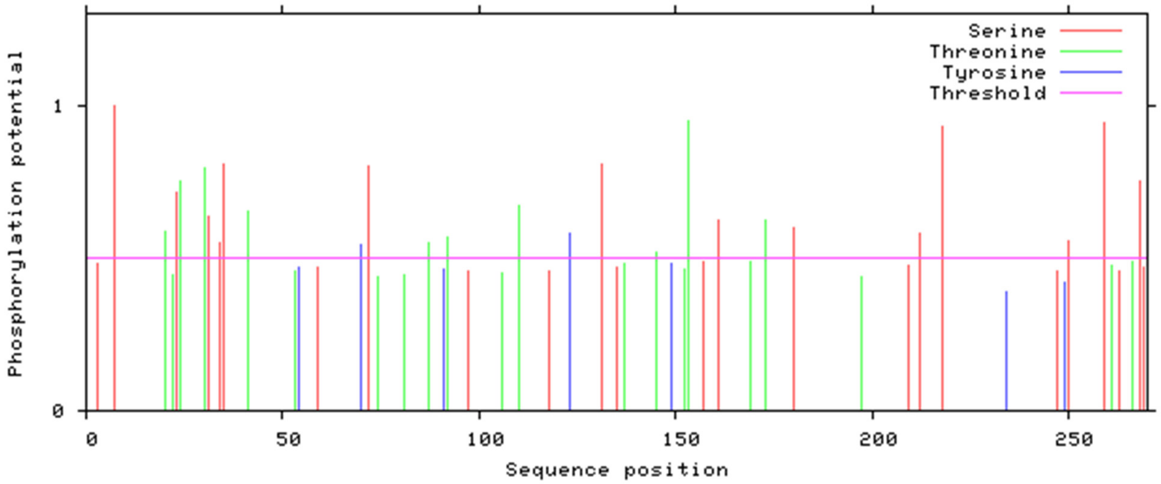

NetPhos 3.1a: predicted phosphorylation sites in CpNIP4 1

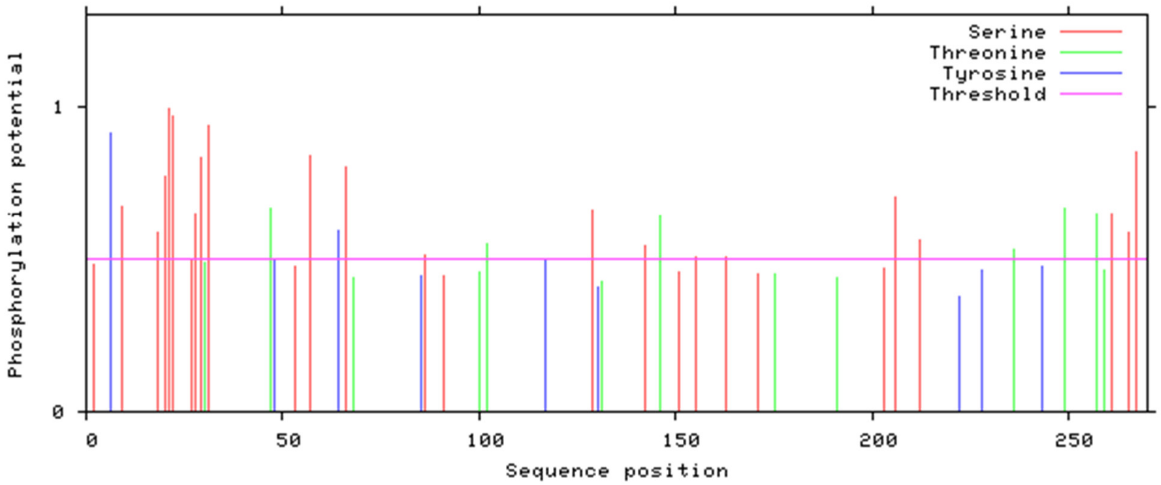

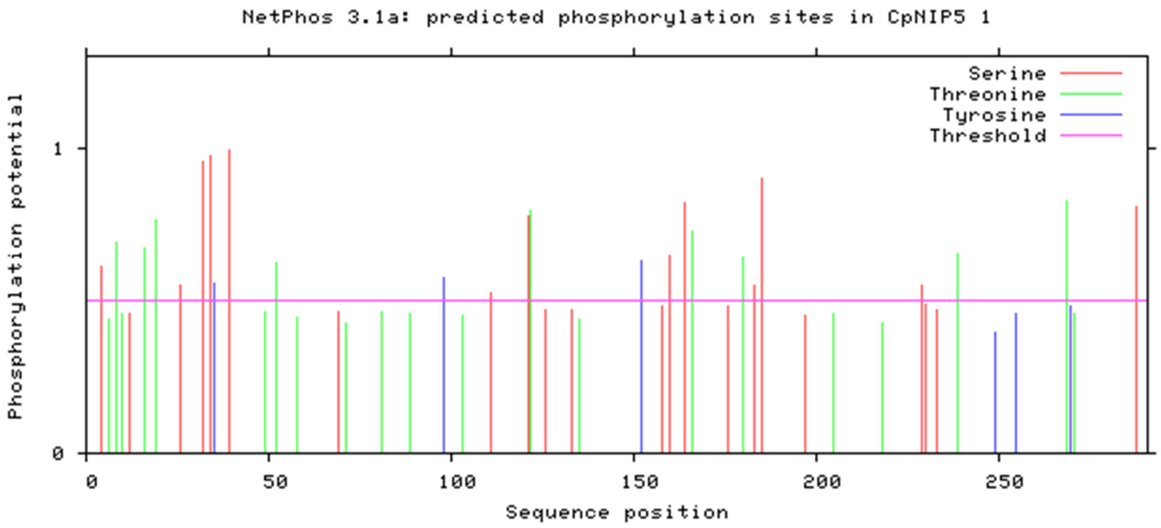
NetPhos 3.1a: predicted phosphorylation sites in CpNIP5 1
Serine
Threonine
Tyrosine
Threshold
Phosphorylation potential
Sequence position
0
1
50
100
150
200
250

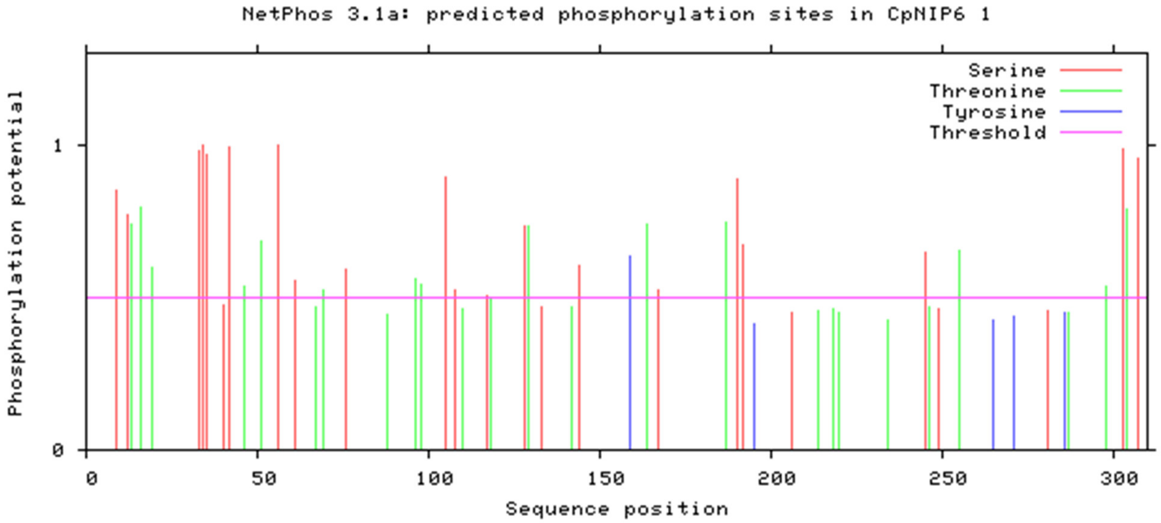
NetPhos 3.1a: predicted phosphorylation sites in CpNIP6 1
Serine
Threonine
Tyrosine
Threshold
Phosphorylation potential
Sequence position
0
1
50
100
150
200
250
300

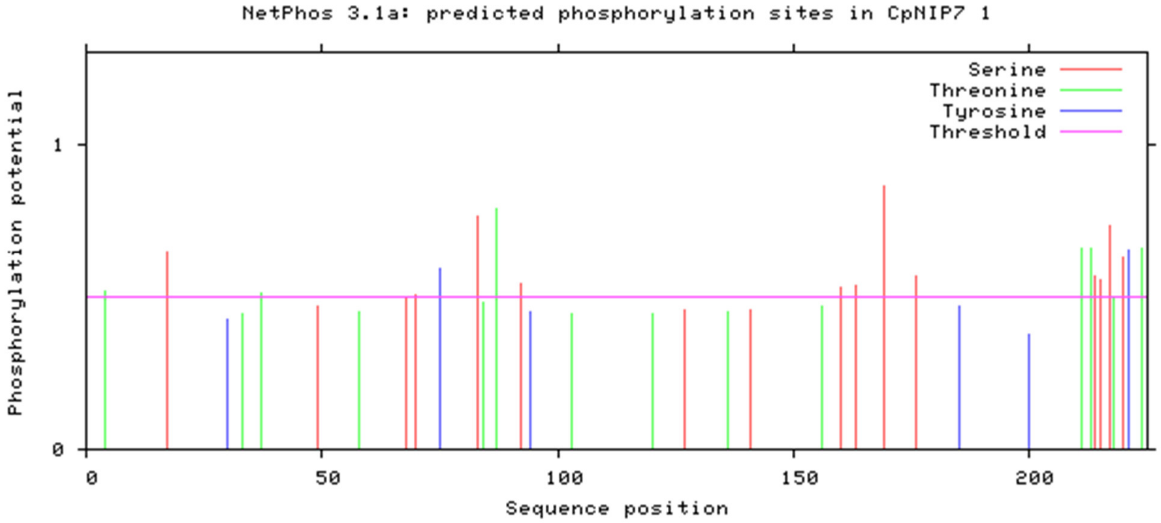
NetPhos 3.1a: predicted phosphorylation sites in CpNIP7 1
Serine
Threonine
Tyrosine
Threshold
Phosphorylation potential
Sequence position
0
1
50
100
150
200

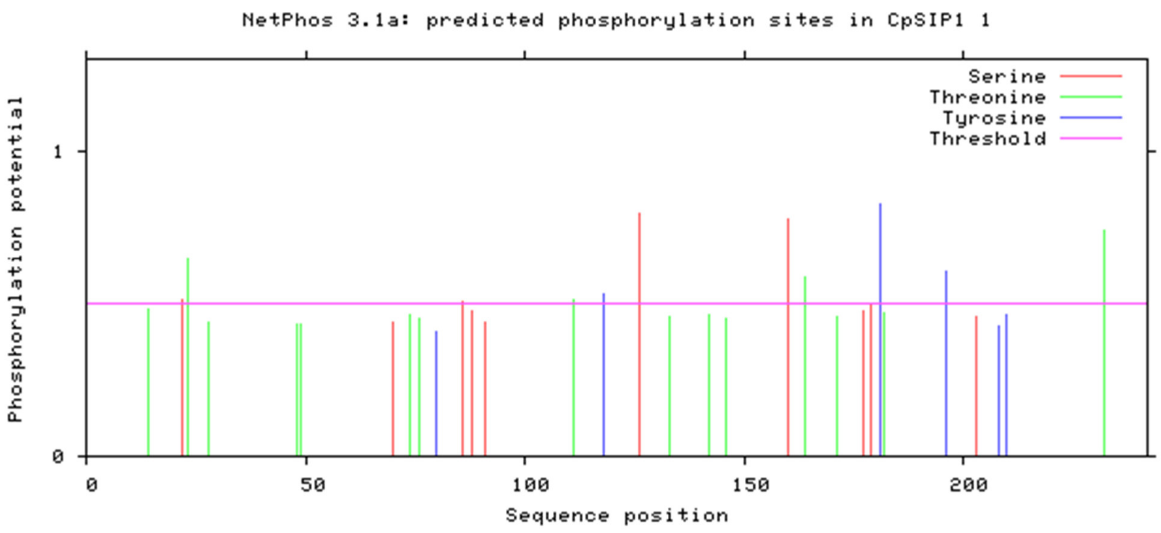
NetPhos 3.1a: predicted phosphorylation sites in CpSIP1 1
Serine
Threonine
Tyrosine
Threshold
Phosphorylation potential
1
0
0
50
100
150
200
Sequence position

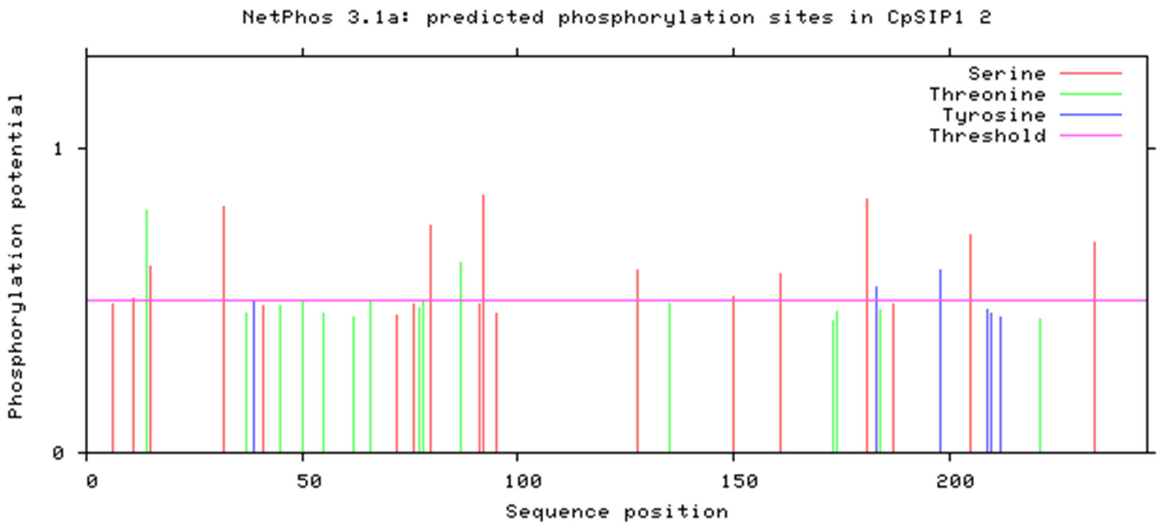
NetPhos 3.1a: predicted phosphorylation sites in CpSIP1 2
Serine
Threonine
Tyrosine
Threshold
Phosphorylation potential
1
0
0
50
100
150
200
Sequence position

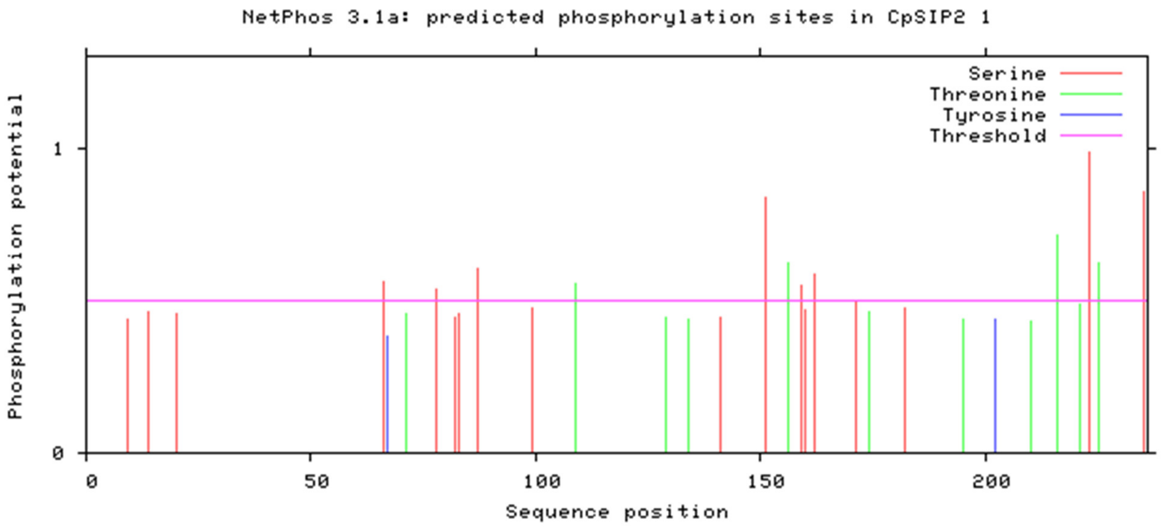
NetPhos 3.1a: predicted phosphorylation sites in CpSIP2 1
Serine
Threonine
Tyrosine
Threshold
Phosphorylation potential
1
0
0
50
100
150
200
Sequence position

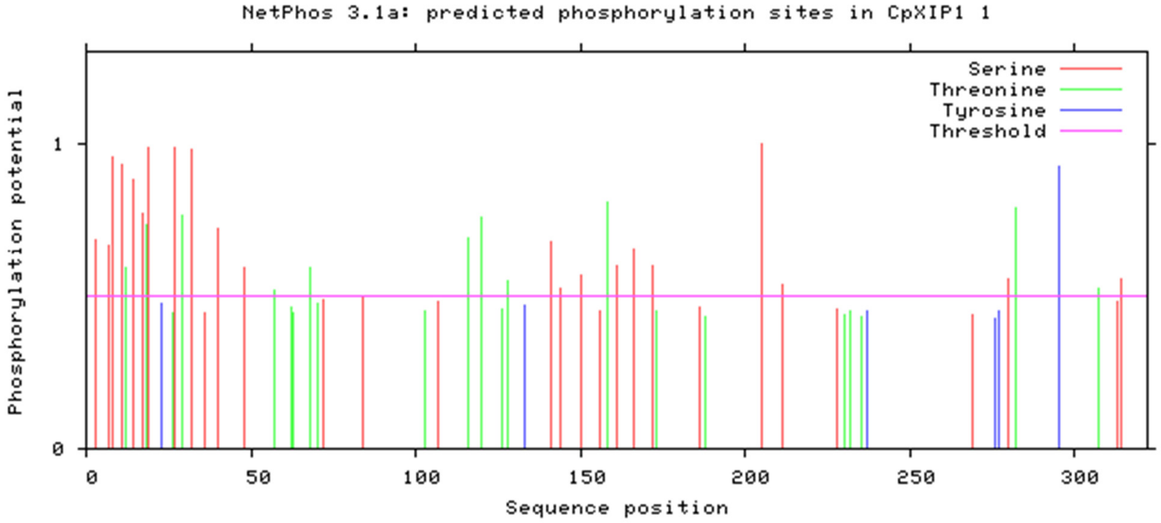
NetPhos 3.1a: predicted phosphorylation sites in CpXIP1 1
Serine
Threonine
Tyrosine
Threshold
Phosphorylation potential
0
1
0
50
100
150
200
250
300
Sequence position

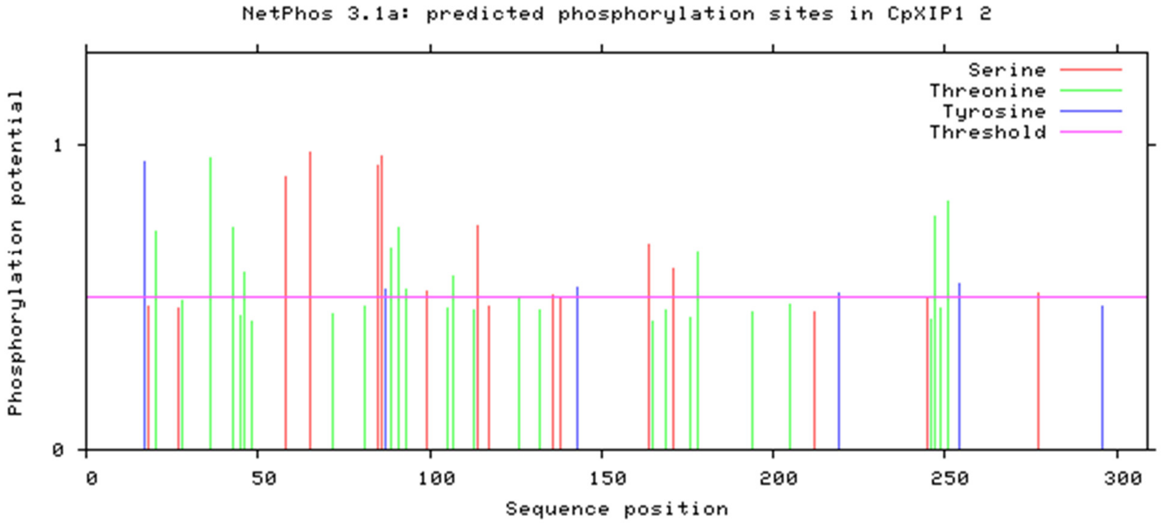
NetPhos 3.1a: predicted phosphorylation sites in CpXIP1 2
Serine
Threonine
Tyrosine
Threshold
Phosphorylation potential
0
1
0
50
100
150
200
250
300
Sequence position
