# Supplementary material for: Genome-Wide Identification and Expression Pattern Profiling of the Aquaporin Gene Family in Papaya (Carica papaya L.)
Source: Int J Mol Sci. 2023 Dec 8;24(24):17276. doi: 10.3390/ijms242417276 (PMC10744249; doi:10.3390/ijms242417276)
Supplement: Supplementary file 1 [file ijms-24-17276-s001.zip › ijms-2706597_Tables S3-S7.pdf]

Table S3 Detailed information of gene secondary and 3D structures of *CpAQPs* gene in papaya

Gene Name	Disorder (%)	α-helix (%)	β-strands (%)	TM helix (%)	Confidence (%)	Alig Coverages (%)
CpPIP1;1	25	59	0	52	100	81
CpPIP1;2	23	60	0	53	100	82
CpPIP1;3	23	59	0	54	100	82
CpPIP1;4	22	60	0	53	100	81
CpPIP2;1	22	63	0	54	100	84
CpPIP2;2	20	59	0	52	100	84
CpPIP2;3	22	64	0	54	100	84
CpPIP2;4	20	62	0	50	100	83
CpPIP2;5	19	62	0	54	100	84
CpTIP1;1	31	55	2	45	100	70
CpTIP1;2	15	69	0	57	100	90
CpTIP1;3	12	70	0	56	100	92
CpTIP2;1	12	68	0	58	100	94
CpTIP2;2	16	71	0	57	100	93
CpTIP3;1	18	67	0	56	100	89
CpTIP4;1	14	70	0	59	100	93
CpTIP5;1	13	70	0	58	100	89
CpNIP1;1	27	59	0	51	100	75
CpNIP2;1	28	60	1	50	100	73
CpNIP3;1	22	61	0	55	100	79
CpNIP4;1	23	63	0	52	100	79
CpNIP5;1	27	60	0	50	100	70
CpNIP6;1	31	60	0	48	100	69
CpNIP7;1	12	65	0	49	100	91
CpSIP1;1	10	72	0	59	100	98
CpSIP1;2	12	71	0	52	100	97
CpSIP2;1	11	73	0	51	100	97
CpXIP1;1	31	57	0	47	100	74
CpXIP1;2	25	60	0	49	100	78

Table S4 Validation results statistics of Ramachandran plot analysis

Name	Residues in most favourated region (%)	Residues in additional allowed region (%)	Residues in generously allowed region (%)	Residues in disallowed region (%)
CpPIP1;1	77.2	19.8	2.5	0.5
CpPIP1;2	78.4	19.1	2.1	0.5
CpPIP1;3	79.4	17.5	2.6	0.5
CpPIP1;4	77.2	19.3	2.5	1.0
CpPIP2;1	78.5	18.0	2.5	1.0
CpPIP2;2	76.4	19.2	3.0	1.5
CpPIP2;3	**91.1**	8.9	0.0	0.0
CpPIP2;4	78.1	17.9	3.1	1.0
CpPIP2;5	76.6	19.3	3.6	0.5
CpTIP1;1	**90.9**	9.1	0.0	0.0
CpTIP1;2	**91.1**	8.9	0.0	0.0
CpTIP1;3	**90.8**	8.7	0.5	0.0
CpTIP2;1	**92.1**	6.8	1.0	0.0
CpTIP2;2	**90.5**	9.0	0.5	0.0
CpTIP3;1	**91.6**	7.4	1.1	0.0
CpTIP4;1	**92.6**	6.3	0.5	0.5
CpTIP5;1	**91.5**	7.4	1.1	0.0
CpNIP1;1	81.4	18.0	0.0	0.5
CpNIP2;1	82.7	17.3	0.0	0.0
CpNIP3;1	80.9	18.0	0.0	1.1
CpNIP4;1	79.1	19.8	0.5	0.5
CpNIP5;1	81.4	18.0	0.6	0.0
CpNIP6;1	82.9	17.1	0.0	0.0
CpNIP7;1	80.3	19.1	0.0	0.6
CpSIP1;1	89.1	9.0	1.0	1.0
CpSIP1;2	86.6	11.4	1.5	0.5
CpSIP2;1	86.3	11.7	1.5	0.5
CpXIP1;1	77.0	16.9	5.2	0.9
CpXIP1;2	**90.0**	8.2	0.9	0.9

Table S5 Accession number for the published RNA-seq data

Sample	Accession
Leaf1	SRR14483186
Leaf2	SRR14483185
Leaf3	SRR14483184
S1	SRR15652208
S2	SRR15652207
S3	SRR15652206
S4	SRR15652205
S5	SRR15652204
S6	SRR15652203
SPHs	SRR14483183
SPHb	SRR14483181
Hy_1	SRR26937493
Hy_2	SRR26937492
Hy_3	SRR26937491
Ec_1	SRR26937486
Ec_2	SRR26937484
Ec_3	SRR26937483
Bud_1	SRR26937489
Bud_2	SRR26937488
Bud_3	SRR26937487

Table S6 Sequencing results statistics of the unpublished RNA-seq data

Sample	Library	Raw Reads	Clean Reads	Raw Base (Gb)	Clean Base (Gb)	Q20 (%)	Q30 (%)	GC Content (%)
PRSV_0_d_1	FRAS210149892-1r	22,119,946	21,211,586	3.32	3.18	98.36	95.04	45.75
PRSV_0_d_2	FRAS210149893-1r	22,419,412	21,561,566	3.36	3.23	98.31	94.93	45.06
PRSV_0_d_3	FRAS210152793-1r	26,577,472	25,512,452	3.99	3.83	98.40	95.17	44.66
PRSV_3_d_1	FRAS210149895-1r	21,002,880	20,111,270	3.15	3.02	98.33	95.07	44.84
PRSV_3_d_2	FRAS210149896-1r	20,509,382	19,660,104	3.08	2.95	98.32	95.06	47.65
PRSV_3_d_3	FRAS210149897-1r	20,263,390	19,439,134	3.04	2.92	98.19	94.77	44.16
PRSV_5_d_1	FRAS210149898-1r	22,190,832	21,254,292	3.33	3.19	98.29	94.94	44.27
PRSV_5_d_2	FRAS210149899-1r	24,328,274	23,109,566	3.65	3.47	98.41	95.18	46.39
PRSV_5_d_3	FRAS210149900-1r	49,326,844	47,485,262	7.40	7.12	97.48	93.14	48.62
PRSV_7_d_1	FRAS210149901-1r	24,172,678	22,983,600	3.63	3.45	98.40	95.15	43.70
PRSV_7_d_2	FRAS210149902-1r	20,113,346	19,326,024	3.02	2.90	98.38	95.08	43.89
PRSV_7_d_3	FRAS210149903-1r	26,922,850	26,011,634	4.04	3.90	98.30	94.88	43.72
PRSV_10_d_1	FRAS210149904-1r	22,622,210	21,797,754	3.39	3.27	98.33	94.94	43.87
PRSV_10_d_2	FRAS210149905-1r	22,023,786	21,248,892	3.30	3.19	98.31	94.90	43.69
PRSV_10_d_3	FRAS210149906-1r	20,285,420	19,479,946	3.04	2.92	98.33	95.00	44.09
Cold_0_d_1	FRAS220294973-1r	21,143,894	20,702,006	3.17	3.11	95.79	89.72	44.87
Cold_0_d_2	FRAS220294974-1r	23,913,944	23,455,072	3.59	3.52	95.82	89.59	44.42
Cold_0_d_3	FRAS220294975-1r	21,075,636	20,801,308	3.16	3.12	95.79	89.59	45.17
Cold_1_d_1	FRAS220294979-1r	21,800,320	21,477,400	3.27	3.22	95.66	89.39	43.46
Cold_1_d_2	FRAS220294980-1r	24,540,796	23,991,376	3.68	3.60	96.11	90.26	44.22
Cold_1_d_3	FRAS220294981-1r	23,117,084	22,570,258	3.47	3.39	95.98	89.99	44.65
Cold_5_d_1	FRAS220294985-1r	23,923,628	23,589,686	3.59	3.54	95.76	89.59	44.82
Cold_5_d_2	FRAS220294986-1r	22,040,650	21,746,454	3.31	3.26	95.95	89.92	44.40
Cold_5_d_3	FRAS220294987-1r	25,596,372	25,288,930	3.84	3.79	95.88	89.80	43.51
Cold_9_d_1	FRAS220294991-1r	21,291,078	20,994,778	3.19	3.15	95.27	88.71	46.60
Cold_9_d_2	FRAS220294992-1r	25,571,852	25,094,388	3.84	3.76	95.42	89.02	44.39
Cold_9_d_3	FRAS220294993-1r	24,694,264	24,225,644	3.70	3.63	95.99	89.93	43.86
Cold_13_d_1	FRAS220294997-1r	23,912,248	23,470,418	3.59	3.52	95.63	89.52	47.41
Cold_13_d_2	FRAS220294998-1r	22,779,552	22,448,056	3.42	3.37	95.92	89.84	43.61
Cold_13_d_3	FRAS220294999-1r	24,614,866	24,260,308	3.69	3.64	95.83	89.66	44.15
seed_1_1	FRAS220260574-1r	20,832,602	20,563,464	3.12	3.08	96.96	91.66	43.52
seed_1_2	FRAS220260513-1r	19,894,608	19,474,370	2.98	2.92	97.07	91.92	43.41
seed_1_3	FRAS220260514-1r	22,801,576	22,535,448	3.42	3.38	97.14	92.01	43.13
seed_2_1	FRAS220260511-1r	24,748,954	24,165,396	3.71	3.62	97.07	91.96	43.06
seed_2_2	FRAS220260512-1r	23,978,520	23,684,280	3.60	3.55	97.04	91.84	41.98
seed_2_3	FRAS220260575-1r	22,910,672	22,626,808	3.44	3.39	96.88	91.50	43.28
seed_3_1	FRAS220260509-1r	22,059,110	21,646,606	3.31	3.25	96.94	91.62	42.70
seed_3_2	FRAS220260510-1r	22,971,210	22,585,950	3.45	3.39	96.57	90.82	42.56
seed_3_3	FRAS220260576-1r	20,994,322	20,761,110	3.15	3.11	96.81	91.38	43.28
seed_4_1	FRAS220260508-1r	20,209,764	19,586,334	3.03	2.94	97.15	92.13	42.21
seed_4_2	FRAS220260577-1r	21,758,874	21,539,536	3.26	3.23	96.84	91.49	43.71
seed_4_3	FRAS220260527-1r	20,103,090	19,793,046	3.02	2.97	97.13	92.30	44.68

Table S7 Primers used in the qRT-PCR analysis of the selected *CpAQPs* gene

Gene name	Primer sequences 5′–>3’
CpPIP2;1-F	AGCCGAATTTGTTGCCACTC
CpPIP2;1-R	TCGCCGCCTTGATTTTTGTC
CpTIP2;1-F	ATGCATGGCAATTCCGATCC
CpTIP2;1-R	ACACGGTGTAAACCAATGCG
CpTIP2;2-F	TGCCATTGCTTTTGGGAAGG
CpTIP2;2-R	TCCGACAAACAGTGCAAACG
CpActin2-F	TTTCCAAGGGTGAGTATGATGAG
CpActin2-R	ACACAGGACACAAAAGCCAACTA
